# Digital Sequence Information and the Access and Benefit-Sharing Obligation of the Convention on Biological Diversity

**DOI:** 10.1007/s11569-023-00436-3

**Published:** 2023-03-28

**Authors:** Frank Irikefe Akpoviri, Syarul Nataqain Baharum, Zinatul Ashiqin Zainol

**Affiliations:** 1 Faculty of Law, Universiti Kebangsaan Malaysia, Bangi, Malaysia; 2 Institute of Systems Biology, Universiti Kebangsaan Malaysia, Bangi, Malaysia

**Keywords:** Digital sequence information, Genetic resources, Access and benefit-sharing, Convention on Biological Diversity

## Abstract

With the advent of synthetic biology, scientists are increasingly relying on digital sequence information, instead of physical genetic resources. This article examines the potential impact of this shift on the access and benefit-sharing (ABS) regime of the Convention on Biological Diversity (CBD) and the Nagoya Protocol. These treaties require benefit-sharing with the owners of genetic resources. However, whether “genetic resources” include digital sequence information is unsettled. The CBD conceives genetic resources as genetic material containing functional units of heredity. “Material” implies tangibility, and for some scholars, “functional units of heredity,” undefined in both treaties, mean full-coding sequences. This article argues that digital sequence information obtained from physical genetic resources, full-coding or not, should be treated as genetic resources. Literal construction of the CBD risks eroding its usefulness and the ABS regime. This is because through bioinformatics, sequence information can easily be obtained from genetic resources for utilization, without physically moving them or concluding ABS agreement with owners. The CBD must evolve with scientific progress also because sequence functionality depends on the state of knowledge. These arguments are vindicated by domestic ABS laws equating genetic information with genetic resources; Nagoya Protocol provisions deeming research exploiting the genetic composition of genetic resources as utilization of genetic resources; and CBD provisions requiring the sharing of benefits from the utilization of genetic resources. Moreover, treaty interpretation and case law demand that generic, scientific terms, such as “genetic resources” and “functional units of heredity” be interpreted in an evolutionary manner to capture scientific developments.

## Introduction

Synthetic biology, which is a novel technology for the de novo design and creation of biological parts, devices, and systems, as well as the modification of regular organisms ([1], p. 6), addresses numerous needs across the scientific and industrial domains, ranging from agriculture, renewable energy, biodiversity conservation to disease prevention, diagnosis, and treatment. This technology relies increasingly on digital sequence information obtained from physical genetic resources and stored in databases that are openly accessible worldwide ([2], pp. 1, 5; [3], p. 1) posing a challenge to existing legal regimes ([4], pp. 43–44; [5], p. 7). A key issue is whether digital sequence information falls within the coverage of the access and benefit-sharing (ABS) regime established under the Convention on Biological Diversity 1992 (CBD), as well as the Nagoya Protocol on Access to Genetic Resources and the Fair and Equitable Sharing of Benefits Arising from their Utilization to the Convention on Biological Diversity 2010 ([6], pp. 102, 260). The ABS regime strives for orderly and just access to genetic resources by requiring users to obtain the prior informed consent of resource owners and conclude equitable benefit-sharing agreements with them.

However, a prominent question continues to be whether genetic resources and the ABS regime of the CBD cover digital sequence information, given its intangibility. Under the CBD, genetic resources mean genetic “material” containing “functional units of heredity.” Here, “material” suggests tangibility, and although the CBD and the Nagoya Protocol do not define functional units of heredity, scholars relying on CBD’s negotiating history view them as gene-coding sequences ([7], p. 6). The CBD and the Nagoya Protocol were directed at tangible genetic resources; neither instrument deals explicitly with synthetic biology ([8], p. 13). This article examines whether digital sequence information derived from regular genetic resources and used in synthetic biology research, is covered by the CBD and its ABS requirements. Synthetic biology and genome sequencing are of special interest to the CBD because of their potential impact on the full realization of its ABS objective. Recent advances in sequencing technologies and the advent of digital sequence information allow parties to use genetic resources without accessing the actual physical genetic material ([9], p. 1105; [10], p. 1). It is, therefore, possible to evade ABS obligations to indigenous peoples and local communities for the use of their genetic resources. Apart from the ABS objective, genome sequencing has implications for the CBD’s other objectives of the conservation of biological diversity and the sustainable use of its components.

There is presently a dearth of scientific and legal analyses of the use of digital sequence information in research and development and the implications this may have for ABS obligations established under the CBD and the Nagoya Protocol ([11], p. 9). There is, in fact, no agreement on whether existing international and domestic ABS regimes cover or should cover the use of digital sequence information and associated benefits. A sound majority of parties to the CBD and the Nagoya Protocol do not even have domestic measures specifically addressing the use of digital sequence information ([11], p. 27). As states are beginning to learn that technological advances may permit the use of their genetic resources through digital sequence information, they have started to ponder this issue ([11], p. 9). Yet, many of them, particularly in Africa, Asia, and Oceania, which are intent on adopting new ABS systems or adapting existing ones to cover the use of digital sequence information, lack proper grasp of recent technological developments, the nature of digital sequence information, and the implications of regulating or not regulating it in their domestic ABS measures. Others are simply reluctant to adopt any measures until there is an international agreement on the matter ([11], pp. 27–28). 

Further, genome sequencing raises concerns for synthetic biologists and other users of genetic resources about the precise scope of ABS obligations facing them ([3], pp. 27, 37). Breaches of domestic ABS laws could expose them to fines, imprisonment or denial of valuable intellectual property protection.^1^ For example, in 2019, the Indian Patent Office suspended the processing of a Patent Cooperation Treaty application because of the unauthorized use of the genetic sequence of an Indian strain of *Plasmodium vivax* ([11], pp. 54–55).^2^ South Africa similarly requires compliance with its ABS law as a condition for the processing and approval of intellectual property applications ([11], p. 20).^3^

Aware of the above challenges, the Conference of the Parties to the CBD (COP) in Decision CBD XIII/16 taken in Cancun, Mexico, at its thirteenth meeting in December 2016 ([12], p. 1), established an Ad Hoc Technical Expert Group (AHTEG). This was meant to address the potential implications of the use of digital sequence information on genetic resources for the CBD’s three objectives of the conservation of biological diversity, sustainable use of its components, and the fair and equitable sharing of benefits arising from the use of genetic resources.

Acting on its mandate under COP Decision XIII/16 of 2016, AHTEG convened in February 2018 to discuss the nexus between digital sequence information and terms used in the CBD and the Nagoya Protocol ([13], p. 2). There was disagreement over whether such information comes within the concept of “genetic resources” in the CBD,^4^ as well as the meaning of “functional units of heredity.”^5^ In July 2018, the CBD’s Subsidiary Body on Scientific, Technical and Technological Advice (SBSTTA) considered AHTEG’s report and issued a recommendation on digital sequence information on genetic resources ([14], pp. 1–4). In November 2018, that recommendation was considered during the fourteenth meeting of COP (COP-14) ([15], pp. 1–3) and the third Conference of the Parties serving as the Meeting of the Parties to the Nagoya Protocol (COP-MOP3) in Sharm El-Sheik, Egypt. At COP-14, two major decisions were adopted, first, Decision14/20 on digital sequence information on genetic resources ([15], p. 1) and second, Decision 14/34 on a comprehensive and participatory intersessional process in readiness for the Post-2020 Global Biodiversity Framework ([16], p. 1).^6^ In preparation for COP-15, Decision14/20 established a science and policy-based process on digital sequence information.

As part of that process, parties and other stakeholders were invited to submit views on whether and how domestic ABS systems address digital sequence information on genetic resources, including the sharing of benefits from their commercial and non-commercial uses.^7^ In addition, there was the establishment of an extended AHTEG to progress work on digital sequence information on genetic resources,^8^ as well as a mandate for CBD’s Executive Secretary to synthesize submissions,^9^ and commission four peer-reviewed studies on the concept, scope and current uses of digital sequence information on genetic resources^10^; traceability of digital sequence information^11^; public and private databases of such information, including terms of access^12^; as well as how domestic ABS regimes address the sharing of benefits from their commercial and non-commercial uses.^13^ Those studies are to guide the work of AHTEG, which is to review them, as well as the synthesis of views and submit findings for consideration by an Open-ended Working Group (WG2020) established under Decision14/20 to consider the global biodiversity framework.^14^ The latter is to submit recommendations to COP-15 on how to address digital sequence information on genetic resources in light of the Post-2020 Global Biodiversity Framework.^15^

In early 2020, the required CBD-commissioned studies were published namely: “Digital sequence information on genetic resources: concept, scope and current use,” (Study 1) ([17], p. 1); “Combined study on digital sequence information in public and private databases and traceability,” (Study 2/3) ([18], p. 1)^16^; and “Fact-finding study on how domestic measures address benefit-sharing arising from commercial and non-commercial use of digital sequence information on genetic resources and address the use of digital sequence information on genetic resources for research and development,” (Study 4) ([11], p. 1). Earlier in 2018, pursuant to Decisions CBD XIII/16 and Nagoya Protocol 2/14, another CBD-commissioned study by Laird and others entitled, “A fact finding and scoping study on digital sequence information on genetic resources in the context of the Convention on Biological Diversity and Nagoya Protocol” was published ([4], p. 1). Between 17 and 20 March 2020, AHTEG held virtual meetings to consider the four CBD-commissioned studies and their implications ([19], p. 2).

Owing to the coronavirus pandemic, informal, virtual meetings continued for most of 2021. Among them were Part 1 of the twenty-fourth meeting of the SBSTTA (SBSTTA-24, May–June) to address, *inter alia,* scientific and technical matters concerning the global biodiversity framework and synthetic biology; Part 1 of the third meeting of WG2020 (WG2020-3, August–September) to negotiate the first draft of the global biodiversity framework and consider feedback from AHTEG on how to reconcile the disparate positions on the sharing of benefits from the utilization of digital sequence information on genetic resources; and Part 1 of the fifteenth meeting of COP (COP-15, October), with some in-person participation, leading to adoption of the Kunming Declaration ([20, 21], pp. 2–3).

In-person meetings, that is, Part 2 of SBSTTA-24 and Part 2 of WG2020-3, among others, resumed from 14 to 29 March 2022 in Geneva, Switzerland, to prepare the groundwork for the adoption of the post-2020 global biodiversity framework during Part 2 of COP-15 scheduled for Montreal, Canada, in December 2022. Numerous recommendations and decisions were made for consideration by COP-15. There was consensus on the way forward and intersessional work on digital sequence information, as well as on indicators and targets for the global biodiversity framework, with agreement to hold a fourth meeting of WG2020 (WG2020-4) in June 2022 in Nairobi, Kenya ([20, 21], p. 24).

Going back to COP-14, there were conflicting views on the applicability of the ABS regime to digital sequence information. The African Union, the African Group, the Group of Like-Minded Megadiverse Countries, and the International Indigenous Forum on Biodiversity all insisted that such information is within the ambit of the international ABS regime [12]. By contrast, Japan and Switzerland argued that the regime exclusively covers tangible genetic resources [22]. New Zealand, the Republic of Korea, the EU, and the World Health Organization (WHO) emphasized the importance of digital sequence information and access thereto for scientific research, global public health, and the conservation and sustainable use of biodiversity [22]. They maintained that public databases and unrestricted access to them are important means of benefit-sharing [22].

Deliberations at AHTEG’s meeting, which informed the SBSTTA’s recommendation, exhibited similar polarity of views that highlights the continuing controversy over the applicability of the CBD and the Nagoya Protocol to digital sequence information. The CBD-commissioned studies on digital sequence information further reiterate the problem posed by digital sequence information for CBD objectives, particularly benefit-sharing, calling for more studies to explore the topic. Without rules or clarity in rules for the sharing of benefits from the use of digital sequence information on genetic resources, provider states may restrict access to such resources, even for non-commercial research that supports biodiversity conservation. Also, as some states^17^ have indicated, absent viable benefit-sharing measures, providers cannot capture monetary benefits from the use of digital sequence information ([11], p. 25).

The present article contributes to efforts aimed at resolving the issue raised by digital sequence information for the CBD and enhancing clarity for the adoption or modification of domestic ABS regimes. The article advances the central thesis that digital sequence information obtained from regular genetic resources should be treated in like manner as physical equivalents and, therefore, considered as genetic resources under the CBD. A rigid construction of the CBD excluding digital sequence information, full-coding or otherwise, from the meaning of genetic resources due to intangibility, would undermine the ABS obligation of the CBD. Although the ultimate aim of synthetic biologists is to design their own sequences ([17], p. 22), current synthetic biology research still relies on digital sequence information derived from regular genetic resources, instead of the physical molecules. Through bioinformatics, sequence information can be extracted from regular genetic resources, without physically moving them or obtaining the consent of their owners and sharing benefits with them. Synthetic biology would seem then to undermine the third objective of the CBD ([23], p. 12,404). An evolutionary interpretation of the CBD that follows scientific progress is necessary also because appreciation of genetic resources and sequence functionality is relative to the state of knowledge.

To buttress the above arguments, this article relies on domestic ABS laws, as well as expert reports that consider digital sequence information as genetic resources; Nagoya Protocol provisions that deem research and development exploiting the “genetic and/or biochemical composition of genetic resources” as the utilization of genetic resources; and CBD provisions requiring equitable sharing of benefits arising from the utilization of genetic resources. Significantly, reference is made to treaty interpretation and case law, including judicial, as well as quasi-judicial decisions. These latter sources require that vague, generic, and technical terms, such as “genetic resources” and “functional units of heredity” be given evolutionary interpretations in light of unfolding scientific developments.

Following this introduction, the article examines the meaning, nature, and importance of genetic sequence information. Next, it analyzes the objectives and conceptual premises of the CBD and the Nagoya Protocol. Also examined here are notions of biological and genetic resources, functional units of heredity, actual or potential value, utilization of genetic resources, and access to genetic resources. Building on these, as well as CBD deliberations, recent commissioned studies and peer-reviewed articles on digital sequence information, this article provides commentary on the problem at hand. It substantiates the argument that rigidly interpreting the CBD to exclude digital sequence information, full-coding or not, from the meaning of genetic resources due to intangibility, would undermine the ABS regime. The article concludes by recapping key points of the discussion and highlighting the need for formal clarification on the meaning and scope of digital sequence information, and particularly, whether the ABS regime applies to it.

## Meaning, Nature, and Importance of Digital Sequence Information

An examination of the meaning, nature, and importance of digital sequence information in synthetic biology research is a useful starting point to provide the requisite technical grounding for the analysis of the concept and its potential impact on the ABS system.

### Meaning

Study 1, which is one of the four studies commissioned by the CBD on digital sequence information on genetic resources pursuant to Decision 14/20, attempts to decompose the concept of digital sequence information into its constituent elements, “digital,” “sequence,” and “information.” Quoting the Oxford English Dictionary, the Study explains that “digital” speaks “of signals, information, or data: represented by a series of discrete values (commonly, the numbers 0 and 1), typically for electronic storage or processing.” This means that digital connotes data held “in computer memory or data storage” ([17], p. 40). “Sequence” suggests “the following of one thing after another in succession” ([17], p. 40). Related to the field of biochemistry, this term is applicable to DNA/RNA nucleotides and the amino acids of proteins, which can be represented in the form of sequences of letters or sets of letters ([17], p. 40). Since material stored on a computer usually takes the form of “a sequence, such as 001,100,100,” it would qualify as digital sequence ([17], p. 40).

More challenging is the element, “information,” which may be distinguished from “data.” The latter describes the intrinsic properties of objects, while the former applies to the meaning or knowledge produced through “cognitive processes applied to such data” ([3], p. 6; [17], p. 31). Other simple definitions proffered for data range from “plain sequence,” “exact sequence of nucleotides,” to “sequences which have not been annotated” ([3], p. 44). Some relate nucleic acids to data, and the result of sequencing to information ([3], p. 45). For others, data becomes information only after analysis, or analysis and interpretation, or the identification of functions ([3], p. 46). However, in practice, it is difficult to differentiate between both terms, such that they are often used interchangeably ([3], p. 46; [17], p. 31).

Beyond the above effort, there is still no agreement on the precise meaning and scope of the concept of digital sequence information, introduced in Decisions CBD XIII/16 and Nagoya Protocol 2/14, which were respectively adopted at the thirteenth meeting of COP and the second meeting of COP-MOP in December 2016 ([4], p. 8; [24], p. 4). At issue is whether, as far as genetic resources are concerned, the concept refers exclusively to data, such as a DNA or RNA sequence, or also extends to amino acid sequences of proteins and the metabolites produced through biosynthesis; whether it also covers processing activities, such as genome assembly, functional analysis, and annotation performed with such data to produce information ([17], pp. 30–31).

The study by Laird and others indicates that the uncertainty surrounding digital sequence information also permeates policy processes at the World Intellectual Property Organization (WIPO), the International Treaty on Plant Genetic Resources for Food and Agriculture (ITPGRFA), WHO’s Pandemic Influenza Preparedness (PIP) Framework, and the United Nations Convention on the Law of the Sea on the Conservation and Sustainable Use of Marine Biological Diversity of Areas beyond National Jurisdiction. The last three respectively use the alternative terms, “sequence data,” “resources in silico,” “digital sequence data,” and “genetic sequence data” ([4], p. 8).

Between 2017 and 2018, parties to the CBD and the Nagoya Protocol, as well as other stakeholders, had the opportunity to express views on how uses of digital sequence information on genetic resources would likely impact the three objectives of the CBD ([17], p. 30). This was in addition to the 2018 Laird and others’ study commissioned by the CBD’s Executive Secretary. Those contributions were summarized by AHTEG on digital sequence information on genetic resources and submitted to COP-14 in November 2018 ([17], p. 30). Despite those efforts, the meaning and scope of digital sequence information remain elusive. Thus, as reiterated in COP Decision 14/20, the concept is presently only being used as a placeholder until there is consensus on a suitable alternative term ([15], p. 1).^18^

Nevertheless, the report of the first (2018) AHTEG on digital sequence information on genetic resources attempts a classification of subject matter that may qualify as digital sequence information and be relevant to CBD objectives. This includes the following: “(a) nucleic acid sequence reads and the associated data; (b) information on the sequence assembly, its annotation and genetic mapping. This information may describe whole genomes, individual genes or fragments thereof, barcodes, organelle genomes, or single-nucleotide polymorphisms; (c) information on gene expression; (d) data on macromolecules and cellular metabolites; (e) information on ecological relationships, and abiotic factors of the environment; (f) function, such as behavioral data; (g) structure, including morphological data and phenotype; (h) information related to taxonomy; (i) modalities of use” ([13], p. 30).^19^

The breath of the above classification further indicates the lack of consensus on the exact scope of digital sequence information ([17], p. 31). In the absence of agreement among AHTEG members, that classification was not adopted by COP-14, robbing it of any binding effect on CBD Parties ([18], p. 13). To improve upon that classification and better clarify the meaning of digital sequence information, Study 1, which builds on the study by Laird and others, proposes four new categories of subject matter that may be covered by the term, digital sequence information. These are: “Group 1—Narrow: concerning DNA and RNA; Group 2—Intermediate: concerning (DNA and RNA) + proteins; Group 3—Intermediate: concerning (DNA, RNA and proteins) + metabolites; Group 4—Broad: concerning (DNA, RNA, protein, metabolites) + traditional knowledge, ecological interactions, etc.” ([17], p. 32).

Study 2/3, another of the four CBD-commissioned studies, which focuses on traceability and databases, adopts a definition that combines (a) and (b) of AHTEG’s classification of digital sequence information presented above. It uses the term, “nucleotide sequence data,” and considers this to mean “ (a) nucleic acid sequence reads and the associated data” and “ (b) information on the sequence assembly, its annotation and genetic mapping” ([18], p. 14). The inclusion of (b) is based on the fact that, in most cases, the submission of nucleotide sequence data to databases is preceded by assembly and annotation. The Study then refers to items (c) to (i) of AHTEG’s classification as “subsidiary information” in respect of which traceability is infeasible, or at least, more difficult than for (a) and (b).

With regard to the classification provided by Study 1 above, it is evident that each group is determined by the degree to which data or information is disconnected from the physical genetic resource. The nearer it is to the originating genetic resource, the narrower the scope of subject matter recognized as digital sequence information and vice versa. For example, Group 1, which is the closest to the originating genetic resource, has a narrow scope, covering only nucleotide sequence data relating to transcription. Group 2, which is intermediate in coverage, covers not only nucleotide, but also protein sequence data, that is, data and information relating to both transcription and translation. Group 3 is also intermediate in scope, but has a broader coverage than Group 2 because, in addition to nucleotide and protein sequence data, it also covers metabolites and biochemical pathways. Thus, it consists of information relating to not just transcription and translation, but also biosynthesis. Group 4, which is the farthest from, and least traceable or even non-traceable to the originating genetic resource, has the widest coverage that includes information concerning not only transcription, translation, and biosynthesis, but also “subsidiary information” like “behavioral data, information on ecological relationships and traditional knowledge” ([17], p. 32).

Other terms, such as nucleotide sequence data, genetic resource sequence data, and genetic sequences used respectively by Study 2/3, the International Chamber of Commerce, and the PIP Framework, correspond with Group 1, which is the narrow category of digital sequence information proposed by Study 1. The March 2020 report of AHTEG on digital sequence information on genetic resources maintains that Groups 1–3 may be considered as digital sequence information, to the exclusion of Group 4, including traditional knowledge, which is viewed as “associated information,”^20^ alternatively called “subsidiary information”.

With the narrowness of Group 1, it is much easier to trace the origin of digital sequence information; whether it is derived from or independently of the utilization of a tangible genetic resource ([17], pp. 32–33). Other than nucleotide and perhaps protein sequence data (Group 2), tracing “subsidiary information” to genetic resources poses a greater challenge ([18], p. 14). Thus, where traceability is important, a broad definition of digital sequence information would be problematic.

Moreover, as explained later in this article, most submissions to the International Nucleotide Sequence Database Collaboration (INSDC), the dominant open access database for genetic sequences, are in the form of nucleotide sequence data ([18], p. 14). Generally, nucleotide sequence data, as used by Study 2/3, result directly from the “nucleotide (DNA or RNA) sequencing of a genetic resource” and this direct connection between sequence data and a genetic resource makes traceability possible. Group 1 is also the basis and starting point for every activity in the other groups and, therefore, likely to cover all uses. Those diverse activities, technologies, or applications rely on information falling within this group. It is estimated that over 55,000 companies across the world use nucleotide sequence data to varying degrees ([18], p. 38). Overall, Group 1 may be said to cover raw sequence data obtained from physical genetic resources. In this context, Study 1 does not discriminate against partial or non-coding sequences, adding that, since non-coding genetic sequences may still perform a useful function in transcription, translation, or biosynthesis, it would be illogical to distinguish them from coding ones.^21^

Another issue may be raised here in respect of CRISPR (clustered regularly interspaced short palindromic repeats) genome editing and how the CBD might deal with edited DNA; how easy it would be to trace an edited gene to the original version. A relevant question would be how similar DNA sequences have to be to their natural source so as to be recognized as having originated from states other than where they were edited, also bearing in mind that they may be modified into unnatural sequences through synthetic biology ([25], pp. 42–43, 53, 63). With such human interference, would the resulting sequences still be considered as natural or human-made? The issue regarding the scope of digital sequence information is picked up again in later sections of this article, which discuss the applicability of the ABS regime of the CBD to digital sequence information, including partial or non-coding sequences.

### Nature and Importance

This section provides a technical background for the understanding of the nature of digital sequence information, as well as its practical importance in science and industry. It relies on the central dogma of molecular biology to explain the structure of the deoxyribonucleic acid (DNA) contained in the nucleus of eukaryotes, how this is transcribed, then translated into proteins, and eventually biosynthesized into metabolites. The DNA comprises four nitrogenous bases called adenine, thymine, guanine, and cytosine (A, T, G, C), with the As being equal in number to the Ts, and the Gs to the Cs ([26], p. 703).

The DNA has two strands held together to form a double helix. Both strands comprise chemical subunits called nucleotides, each of which has a deoxyribose sugar, a phosphate group, and a nitrogenous base (A, T, C, or G) ([17], p. 13). The deoxyribose sugar and phosphate groups constitute the backbone of each strand to which the nitrogenous bases are attached. Generally, an A base on one strand always bonds with a T base in the opposite strand, just as a G base on one strand always bonds with a C base in the opposite strand ([11, 17], p. 13). The order in which these bases are arranged in a DNA strand is called the DNA sequence ([18], p. 14).

Also, based on the central dogma, the DNA regulates the creation of another type of nucleic acid called ribonucleic acid (RNA). The latter regulates the creation of proteins, which determine the structure of cells and the way they function ([17], p. 13). The DNA stores instructions for the production of these proteins. Through a process called transcription, an enzyme known as RNA polymerase forces its way through minute openings in the membrane of the nucleus, copies information encoded by a DNA sequence, and transfers it into a newly created strand of RNA called messenger RNA (mRNA) ([27], p. 131; [28], pp. 62–64).

The mRNA carries that information to a ribosome in the cell’s cytoplasm where it is converted into an ordered chain of amino acids that form a protein ([29], p. 101; [17], p. 14). This process by which sequence information in RNA is converted into protein is called translation ([17], p. 14). Once proteins have been formed, biosynthetic enzymes convert them into metabolites, such as carbohydrates, proteins, lipids, organic acids, and antibiotic agents ([17], p. 15). This process is known as biosynthesis. The DNA, RNA, proteins, and metabolites are involved in the activities that go on in cells, including growth and reproduction ([24], p. 17). 

Technological advances of the last several decades, starting with Sanger sequencing of the 1970s, have facilitated the sequencing of increased DNA lengths and even whole genomes ([2], p. 1; [3], p. 1). By the 1990s, during the Human Genome Project, it was possible to sequence the human genome comprising about 3 billion base pairs ([17], pp. 17–18). More recent high-throughput next-generation sequencing technologies have further increased the speed and accuracy of sequencing, while also reducing costs drastically. For example, whereas the Human Genome Project, which lasted about 4 years, consumed approximately $US450 million, by 2015, the cost of human genome sequencing had dropped below $US1,500, with efforts still being made to bring this to under $US100 ([24], p. 5).

Concurrently, ease of access to sequencing technologies made possible by increasingly affordable costs soon inundated scientists with vast amounts of sequence data that proved too complex to handle. Bioinformatics and computational biology ameliorated this challenge by providing computational tools and platforms, including freely accessible databases that receive continually rising levels of DNA sequences and functional annotations, which together form what may broadly be considered as digital sequence information ([17, 30], pp. 20–21). These facilities permit the storage, analysis and comparison of sequences, identification of mutations or commercially valuable gene functions ([17], p. 20).

Availability of sequencing tools and platforms for the collection and management of large-scale genomic data, greater understanding of living systems, as well as gene functionality, are complemented by increasingly affordable gene synthesis services ([31], p. 6). Together, these developments permit novel experiments that were previously impossible due to scientific or financial limitations ([32], p. 1391). These activities depend on what may qualify as digital sequence information ([17], p. 11). For example, through bioinformatics, systems and synthetic biology, scientists can now digitally access sequences from genetic resources, without having any contact with physical molecules.

Across the scientific and industrial fields, increasing numbers of technologies and applications that rely on digital sequence information address wide-ranging needs ([10], pp. 1–2). These range from biofuels and industrial chemicals produced using engineered microbes as biofactories ([17], p. 27), to plants engineered for higher productivity and disease resistance ([33], pp. 103, 872; [34], pp. 80–90). Others include the identification and extraction of plant-based medicinal compounds, such as the antimalaria compound, artemisinin, promising cheaper and faster new drug production ([17], p. 27). The importance of digital sequence information can similarly be seen in the area of biodiversity conservation through the discovery and identification of new species aided by longer DNA sequences, DNA barcodes, and sequence databases ([10], pp. 1–2; [35], pp. 1–2).

A particularly transformative technology enabled by digital sequence information is CRISPR, which allows for cheaper, faster, and more accurate modification than previous gene editing molecular tools, including zinc finger endonucleases ([36], p. 20). The technique also aids the design of faster diagnostic tests for disease agents like the Ebola virus, vastly reducing the possibility of infection ([17], pp. 27–28). As a whole, researchers in virtually every area of the scientific field access and utilize genetic sequence information from public databases ([18], p. 28). Simultaneously, as this article demonstrates, these developments unsettle current legal frameworks.

## Convention on Biological Diversity and the Nagoya Protocol

The CBD was adopted in 1992 during the Rio Earth Summit. Alongside the quest for the conservation and sustainable use of biodiversity, this instrument aims to ensure orderly access to genetic resources and the fair and equitable sharing of benefits arising from their utilization.^22^ This last objective serves the interest of states providing genetic resources, especially those in the developing world, which are eager to curb the misappropriation of their genetic resources and related traditional knowledge, as highlighted by *Turmeric* and *Neem*, among other cases.

In *Tumeric*, a patent was granted on the use of the turmeric plant for healing wounds, an already established Indian traditional knowledge. India successfully petitioned the United States Patent and Trade Mark Office (USPTO) to nullify that patent ([23], p. 12,404). Similarly, in *Neem*, patents were obtained for products derived from extracts of the neem tree, which had long been known to and bred by Indian farmers. Following a petition by India, the USPTO and the European Patent Office annulled those patents ([23], p. 12,404). More recently, the availability of gene sequencing software, steady expansion in the sequencing of genetic resources, and open dissemination of sequence information have increased concerns about provider states losing control over their genetic resources. To achieve the objective of fair and equitable benefit-sharing, the international ABS regime was established as a legal framework for orderly and just access to genetic resources and traditional knowledge. Under the CBD, states exercise sovereign rights over biological resources in their territories.^23^ National governments can control access to their genetic resources, based on domestic ABS legislation.^24^ Parties seeking access to such resources must obtain their prior informed consent, including, where necessary, that of local communities.^25^ Access, where granted, should be on mutually agreed terms.^26^ Parties must also agree to share with provider states, in a fair and equitable manner and on mutually agreed terms, results from research and development, and benefits obtained from the commercialization and other utilization of genetic resources.^27^

The CBD does not define “fair” and “equitable.” Presumably, it requires transparency in dealings, accuracy of information, opportunity for informed decisions and benefit-sharing based on each party’s contribution ([37], p. 179). Similarly, mutuality of agreement on the terms of access and benefit-sharing seeks to prevent unfair contract terms that unduly disadvantage one party because of power inequalities ([38], p. 33). Terms should be freely negotiated and agreed between both parties, including the process of access, potential uses and the sharing of resulting benefits ([39], p. 9).

States’ sovereignty over their biological resources and traditional knowledge, including the requirements of prior informed consent and equitable benefit-sharing on mutually agreed terms, are reiterated in the Nagoya Protocol.^28^ This Protocol implements the CBD and applies to genetic resources covered by Article 15 of that Convention.^29^ To effectively implement the ABS regime, states are required under the Nagoya Protocol to ensure that genetic resources utilized in their territories have been accessed in compliance with domestic ABS laws and cooperate with provider states to redress violations.^30^ As of March 2022, the CBD had 196 parties, including European Union (EU) members ([11, 40], p. 2), while the Nagoya Protocol had 133 parties [40]. The U.S. signed the CBD, but did not ratify it. Several parties to the CBD and the Nagoya Protocol have adopted domestic and regional laws implementing the international ABS regime ([41], p. 536). These legislative measures are meant to help states reassert control over their genetic resources, while also manifesting a desire for international cooperation to ensure organized access to those resources.

### CBD’s Conceptual Framework

Since the ABS regime centers on control over “genetic resources”^31^ of “actual or potential value” containing “functional units of heredity,” as well as their “access” and “utilization,” it is important to examine what these terms actually mean. This is because ABS obligations are triggered only when they are established ([42], p. 2).

### Biological and Genetic Resources

According to the CBD, “‘biological resources’ include genetic resources, organisms or parts thereof, populations, or any other biotic component of ecosystems with actual or potential use or value for humanity.”^32^ Genetic resources are “genetic material of actual or potential value,”^33^ while genetic material means “any material of plant, animal, microbiological or other origin containing functional units of heredity.”^34^ It has rightly been observed that these definitions are convoluted, especially for non-biologists ([43], p. 35, and potentially overlapping ([37], p. 34). Organisms are obviously genetic resources, despite the apparent attempt to differentiate between them. Moreover, genetic material is so widely defined that it virtually conflates with biological resources ([44], p. 338). Although genetic resources are a category of biological resources ([45], p. 6),^35^ failure to clearly distinguish between them may render it difficult to determine whether the former, rather than the latter, have been accessed or utilized. Interaction with a whole gamut of resources may, therefore, trigger ABS obligations ([46], pp. 63–64].

Over the years, that lack of precision has caused controversy over whether the international ABS regime applies only to genetic resources or extends to biological resources, as suggested by some domestic ABS laws ([45], p. 6; [47], pp. 62, 72–74).^36^ This raises the question as to the actual difference between both categories of resources. Two perspectives may be advanced. First, biological resources, such as plants and animals, are vital for scientific research and provide ingredients for the production of pharmaceutical, aesthetic, industrial, and other commercial products. In this sense, as long as biological resources contain usable material, they constitute genetic resources, which, according to the CBD, are materials of plant, animal, and microbial origin ([48], p. 282). It is irrelevant, in this case, whether the genetic or biochemical element is exploited.

Second, whether a material is a biological or a genetic resource may be determined on the basis of the intended use ([45], p. 6). A biological material is a genetic resource, if the aim is to exploit its genetic composition or other units of heredity, rather than its physical properties ([49], p. 14). Therefore, if, for example, the biochemical composition of a plant is exploited to produce drugs or a tuber of yam is used as food, that plant or tuber of yam is a biological resource. On the other hand, if genes from the plant are used to make drugs or the tuber of yam is used for breeding, both are genetic resources, and only in this latter scenario would ABS obligations be triggered ([50], p. 17).

The second approach above could also undermine the ABS regime because resources accessed based on claims of non-genetic uses could later be put to genetic uses, thereby evading ABS obligations ([47], pp. 60–64). Emphasis on genetic uses would also exclude many resources used in drug development from the ABS regime, since their biochemical, rather than genetic compositions, are exploited ([17], p. 27).^37^ The Nagoya Protocol’s definition of “utilization of genetic resources,” examined later in this article, indicates that the ABS regime covers material with biochemical, as well as genetic composition ([47], pp. 60, 64–65).

### Functional Units of Heredity

Another key challenge in interpreting the CBD lies in its definition of genetic resources as genetic material containing “functional units of heredity,” without clarifying the quoted term. The Nagoya Protocol only passively refers to the term in Article 2(e), where it defines a derivative as “a naturally occurring biochemical compound resulting from the genetic expression or metabolism of biological or genetic resources, even if it does not contain functional units of heredity.” Attempts to fill this gap rely on CBD’s negotiating history by suggesting that between 1989 and 1992 when the instrument was negotiated, the emphasis was on protein-coding sequences. Impliedly, “functional units of heredity” could only have been intended to mean full-coding sequences ([7], p. 6).

### Actual or Potential Value

It is also vital to examine other key elements in CBD’s definition of genetic resources and genetic material. According to Article 2(10) of the CBD, genetic resources should have “actual or potential value.” Neither the CBD nor the Nagoya Protocol explicitly defines this term. However, according to Preamble 1 of the CBD, such value may be “intrinsic, ecological, genetic, social, economic, scientific, educational, cultural, recreational and aesthetic,” which suggests that value does not only consist in genetic material ([51], pp. 5–6).^38^ In respect of genetic material, some commentators explain that every such material has, at least, a “potential” value ([52], p. 22). Potential value is the unascertained value of a genetic resource, unlike actual value, which is already known. Possession of value indicates that a genetic material is capable of utilization. Therefore, it is the potential for utilization that creates value ([45], p. 7), while utilization activates ABS obligations ([46], p. 52).

### Utilization of Genetic Resources

If the utilization of genetic resources creates ABS obligations, what then is “utilization”? According to Article 2(c) of the Nagoya Protocol, utilization of genetic resources “means to conduct research and development on the genetic and/or biochemical composition of genetic resources, including through the application of biotechnology as defined in Article 2 of the CBD.” According to Article 2 of the CBD and Article 2(d) of the Nagoya Protocol, biotechnology refers to “any technological application that uses biological systems, living organisms or derivatives thereof, to make or modify products or processes for specific use.” Utilization, as defined here, is to be differentiated from non-ABS uses, such as the purchase and consumption of biological resources as commodities, for example, fruits, tubers, or vegetables ([37], pp. 39–40). Nevertheless, if resources meant for trade or consumption are subsequently utilized in research and development, they would activate ABS obligations ([53], p. 12).

### Access to Genetic Resources

An additional question relates to what constitutes “access” to genetic resources. It is unclear from the CBD or the Nagoya Protocol whether, as maintained by some countries,^39^ access occurs simply upon the taking of a tangible genetic material or, as claimed by others,^40^ only when such material is used for a purpose qualifying as the utilization of genetic resources, for example, the exploitation of its genetic composition. Since neither treaty defines “access,” it can mean physically obtaining genetic resources or using them in innovation ([54], p. 43; [55], p. 9). Some commentators suggest that an approach based on utilization, which requires parties seeking access to biological material to disclose the intended use, could provide certainty on access to genetic resources ([42], p. 26). Any use amounting to “utilization of genetic resources” would establish access and warrant ABS obligations ([37], p. 42).

However, that approach could also undermine the ABS regime, either because initially non-genetic uses subsequently develop into genetic uses or intended uses are deliberately misrepresented to avoid ABS obligations. This, perhaps, partly explains the paltry benefit that has, so far, accrued from the ABS regime ([7], p. 16). If, on the contrary, ABS obligations are triggered at the point of utilization, provider states would face difficulty in enforcing such obligations in user states, which are clearly beyond their sovereign control ([7], pp. 16–17). It should be noted that in a state, such as Colombia, “access” and “use” are treated as the same. Thus, the utilization of physical genetic resources or digital sequence information is governed by access contracts that impose benefit-sharing obligations ([11], p. 15). In respect of digital sequence information, Brazil focuses on benefit-sharing, rather than access ([11], p. 16).

### Nagoya Protocol

As already remarked, the Nagoya Protocol is meant to implement the CBD. It is unclear, however, whether the Protocol covers access to genetic resources occurring before or after 12 October 2014, when it became effective. Provider states adopt the former position, but user states favor the latter ([56], p. 32).^41^ Adoption of the latter position would exclude significant amounts of genetic resources previously accessed in provider states, including related benefit-sharing obligations, even if utilization occurs after the Nagoya Protocol’s effective date ([31], pp. 17–18). This would mean that digital sequence information derived from such resources and utilized would not be covered by the ABS regime ([57], p. 19).

On the other hand, treaty law apparently precludes the former position, which could be viewed as retroactive application of the Nagoya Protocol.^42^ Nevertheless, it has been suggested that adoption of the former position would only amount to retroactive interpretation of the Protocol, rather than its retroactive application ([31], p. 18). The Vienna Convention on the Law of Treaties (Vienna Convention) forbids retroactive treaty application, but not retroactive interpretation ([58], p. 137). The difference between treaty application and interpretation is tenuous, and for some, doubtful, but both are distinguishable ([56], p. 32).^43^

Interpretation is the task of ascertaining the actual meaning of a treaty, whereas application is the task of determining the repercussions visited by a treaty on the occurrence of specific facts, acts, or situations ([56], p. 32). The objective of interpretation is to determine the intention of parties from the words of a treaty in the relevant context ([59], p. 365; [60], p. 849).^44^ Once the meaning of a treaty is determined, the next step is to apply it to the facts of the case at hand ([56], p. 32).^45^ This suggests that interpretation is an aspect of treaty application [61]. However, application does not always involve interpretation. The need for interpretation arises where there is ambiguity or vagueness that renders the plain wording of a treaty capable of different meanings or impossible to understand ([60], p. 849). Where the meaning of a treaty is clear, it is applied, not interpreted ([56], p. 32; [62], p. 365). Current international law, as reflected in Article 28 of the Vienna Convention, contains a general presumption against the retroactive application of treaties. A treaty cannot be applied to “acts or facts that occurred or situations that ceased to exist” prior to its effective date, unless a contrary intention is specifically expressed in the treaty or can be implied from its terms. 

In the context of the Nagoya Protocol, to determine its temporal scope, reference must be made to its text, subject matter and nature of obligations imposed. The Protocol’s objective is the fair and equitable sharing of benefits from the utilization of genetic resources.^46^ As earlier mentioned in this article, these resources are those within the scope of Article 15 of the CBD. Access to them shall be with the prior informed consent of providers. Benefits derived from their utilization, including subsequent applications and commercialization, must, in line with the said Article 15, be shared with providers, in a fair and equitable manner, based on mutually agreed terms.

The Nagoya Protocol came into force 90 days after the date on which the fiftieth instrument of ratification was deposited,^47^ which was on 12 October 2014. The principle of non-retroactivity precludes application of the Protocol to genetic resources accessed and utilized before that date. At the same time, it should be noted that the presumption against retroactivity is a rebuttable, not an absolute one. As implicit in Article 28 of the Vienna Convention, a treaty can be applied retroactively, if the parties express such an intention in the treaty or it can be implied from its terms.

More importantly, for the present purposes, although the Nagoya Protocol can conveniently be said to exclude genetic resources accessed and utilized before 12 October 2014, it does not specifically address cases of genetic resources accessed before that date but utilized thereafter. This lack of clarity calls for interpretation, rather than application stricto sensu of the treaty. Interpretation is necessary to determine the intention of the parties as to its temporal scope. In this regard, it bears recalling that, according to Article 3 of the Nagoya Protocol, this Protocol applies to the genetic resources covered by Article 15 of the CBD. That provision of the CBD took effect as far back as 1993 and requires the prior informed consent of provider states before access to and utilization of their genetic resources. It also requires the sharing of results from research and development, as well as benefits accruing from commercial and other utilization of such resources. Therefore, provider states could argue that they are merely interpreting the Nagoya Protocol in a way that would best ensure its effectiveness, as well as that of the CBD, in light of their object and purpose ([39], pp. 72–73), which is the fair and equitable sharing of benefits from the utilization of genetic resources with providers. Article 4(4) of the Nagoya Protocol stresses that the Protocol implements this benefit-sharing objective of the CBD. It is, thus, aimed at rendering the CBD effective, rather than weakening it.

That contention is supported by the principle of “systemic integration,” embodied in Article 31(3)(c) of the Vienna Convention, which views international law as a system. Therefore, it demands that, in applying a particular rule, other rules that are relevant to the matter at hand must also be considered ([63], pp. 13–14).^48^ Relevantly, Article 4(3) of the Nagoya Protocol requires the Protocol to be implemented in harmony with other international instruments relevant to it. The Vienna Convention is arguably one such instrument. There is an even stronger need for the Nagoya Protocol to be implemented in a manner supportive of the CBD, which is the international instrument it is specifically intended to enforce.

The approach suggested above would amount to retroactive interpretation, rather than application of the Nagoya Protocol. It would help to fill a gap that could otherwise allow genetic resources accessed before its effective date, but utilized subsequently, to escape regulation with adverse effects on provider states. Facts or situations that amount to breaches cannot be excluded from regulation, if they continue after a treaty comes into force, otherwise the rights conferred would be rendered illusory and ineffective. Moreover, Article 5(3) of the Nagoya Protocol permits parties to adopt appropriate legislative, administrative, or policy measures to ensure the fair and equitable sharing of benefits derived from the utilization of genetic resources, as required by this Protocol and the CBD. Thus, provider states could stipulate in their domestic ABS measures that all uses of genetic resources made after 12 October 2014 are covered, even if such resources were accessed before that date.^49^

It is equally arguable that, strictly speaking, what is addressed by the Nagoya Protocol is not the accessing of genetic resources per se (act), which is generally permitted, subject to prior informed consent, but doing so in a manner adverse to the interest of providers; that is, the utilization of those resources, without fair and equitable benefit-sharing with providers (effect).^50^ This is the real obligation imposed.^51^ In other words, the utilization of genetic resources without benefit-sharing, rather than the accessing per se, is what causes the adverse effects protected against. States have an obligation to prevent such effects,^52^ which constitute the “wrongful act” precipitated by the preceding action of accessing genetic resources.^53^

Accordingly, the issue as to retroactivity centers on whether the situation causing adverse effects, namely utilization without benefit-sharing, has ceased or still continues after the Nagoya Protocol is in force. Viewed in this sense, Article 28 of the Vienna Convention cannot be said to preclude benefit-sharing obligations on users in respect of genetic resources accessed before the Nagoya Protocol came into force, insofar as their utilization continues after that time. Since Article 28 of the Vienna Convention provides that a treaty does not apply to situations that have ceased to exist, unless a contrary intention is established, the converse is also true that the obligations of a treaty can be extended to situations that have not ceased to exist after the treaty is in force, even if the acts creating them started at a previous time.^54^

Hence, a suggested strategy for overcoming the above dilemma is to subject all new uses of genetic resources occurring after the Nagoya Protocol’s effective date to benefit-sharing obligations, even if those resources were accessed before that date ([57], p. 20). This would afford provider states some benefit from such resources, especially since benefit-sharing under Article 5 of the Nagoya Protocol strictly does not depend on the circumstances of access under Article 6 ([57], p. 20). It should be clarified, for the avoidance of doubt, that where entirely new, human or machine-designed sequences are used, as synthetic biologists contemplate in the bottom-up approach, no benefit-sharing obligations would arise under the CBD, since such sequences are not derived from regular genetic resources ([64], p. 640). Indeed, only the states where such sequences were invented would possibly have any claim. This raises the interesting point of whether natural genomic DNA would be left behind as synthetic biology moves toward completely novel designs. As discussed later in this article, “access” to genetic resources also poses the important question of whether only uses of physical genetic material would constitute access under the ABS system or whether that term also extends to intangible items.

## Assessing the Controversy

The foregoing analysis throws up several interrelated questions that require fuller examination. Specifically, the following questions call attention: (i) given its merely informational, intangible nature, does digital sequence information qualify as genetic material and, therefore, as a genetic resource covered by the CBD and its ABS regime?; (ii) even if it does, could every such information, even if non-coding, be considered as “functional units of heredity”?; (iii) does digital sequence information have value?; (iv) is digital sequence information capable of utilization? and (v) does “access” to genetic resources occur only when there is interaction with a physical genetic material?.

It is evident from the preceding discussion that the CBD defines “genetic resources,” “genetic material,” “derivative,” and even “biotechnology” all in material terms ([65], p. 15). In effect, it considers genetic resources as tangibles. Hence, on a literal interpretation, digital sequence information cannot be covered by the term, “genetic resources” ([66], p. 3; [67], p. 25). Further, as suggested by those relying on CBD’s negotiating history, “functional units of heredity,” refer to full-coding genes. This means “the nucleotides constituting a ‘functional unit’ on nucleic acids,” which again signifies materiality ([37], p. 179). On this account, digital sequence information is apparently excluded from the meaning of genetic resources due to immateriality. Even if it were included, non-coding or partially coding sequences would not be covered since they do not qualify as functional units of heredity.

In interpreting a treaty term, such as “functional units of heredity,” extrinsic aids, including negotiating histories, are useful for resolving uncertainties.^55^ In the present case, however, reliance on CBD’s drafting history is assailable. Despite the contention that genes are the units of heredity, non-coding sequences could also contain functional units that are crucial, for example, to gene expression ([68], p. 9). Further, as pointed out earlier in this article, the CBD’s definition of “derivative,” includes the phrase, “even if it does not contain functional units of heredity.” Therefore, the absence of functional units of heredity does not necessarily rob a genetic material of value ([47], pp. 60, 67–68). Genome sequences that are not full-coding could still be combined for useful functions, including the transcriptional and translational regulation of protein-coding sequences ([69], pp. 449–451; [70], p. 73).

Indeed, synthetic biology research is directed not only at the enhancement of protein-coding sequences, but also those performing other valuable functions. Through such research, parts of nucleic acid smaller than genes have been exchanged between species to synthesize functional gene products ([37], p. 35). According to Muller, with biological parts, natural sequences can also be enhanced to produce pharmaceutical compounds and industrial enzymes ([65], p. 16). This renders them valuable. Consistent with this argument, other experts maintain that biological resources, as defined in the CBD, cover DNA extracts, including a chromosome, gene, and plasmid or any part thereof, including the promotor part of a gene, each of which could have a distinct function ([71], p. 2; [11], p. 8).

Enhancement of sequences encoding proteins or other vital functions involves substantial utilization of physical genetic material ([31], p. 9). Such material is the source of many digital sequences ([53], p. 3; [11], pp. 19, 29) particularly in top-down synthetic biology research. As Garrity and others explain, synthetic biology is facilitated partly by the creation of new digital components based on natural models ([51], p. 19). The existence of companies that synthesize digital sequences is further testimony of their significant value in synthetic biology research, even though intangible ([31, 72], p. 9). Therefore, exclusive focus on physical genetic material or protein-coding ones, has the potential to sidestep the ABS system by freeing a broad array of uses from its coverage ([17], pp. 4, 28, 39).

Emphasis on protein-coding sequences is untenable for other reasons. Genes are structurally mutable. Through “natural genetic engineering” encoded information could be altered by other molecules, such as proteins ([73], p. 5). Moreover, genes alone do not explain the intricacy of heritable features in living systems. The human genome, for example, has only about 25,000–30,000 genes, which cannot account for the far greater range of human traits ([74], p. 2114). The notion of one gene-one protein is obsolete because a single gene may encode several proteins ([43], p. 35; [27], p. 131).

Additionally, appreciation of DNA functionality and potential value is relative, being dependent on the current state of scientific knowledge [75].^56^ Perceived non-coding sequences, the so-called “junk DNAs,” may encode genetic information awaiting discovery ([68], pp. 4, 9); information that may be vital to “the proper genetic expression, replication, transmission and architecture of the genome” ([37], p. 36; [17], pp. 4, 39). Thus, having raised the question whether non-coding sequences should be classified as digital sequence information, CBD-commissioned Study 1, goes on to conclude that “genetic elements which do not encode proteins (such as promotors) may have a natural functional role in transcription, translation or biosynthesis and on this basis may be considered an inherent part of the underlying genetic resource, such that it would be illogical to distinguish between coding and non-coding sequences.” This appreciation of non-coding sequences fits them within subject matter that may qualify as digital sequence information.^57^

Phenomenal advances in the life sciences witnessed in recent decades point vividly to the ever present possibility that “research may identify new functions of genetic resources and streamline the development of technology, new goods and services” ([65], p. 14). For this reason, Muller argues that “actual or potential value,” as used in the CBD, should cover both the known and yet to be discovered value of a genetic resource ([65], p. 14). This ostensibly covers all genetic resources. Hence, it is controversial to restrict “functional units of heredity” to genes ([7], p. 16). Indeed, for some commentators, this term signifies not just genes, but “the sum of all interacting parts on the genome and all physical factors that control their expression and their direct products” ([37], p. 36).

As previously mentioned in this article, a genetic resource should have actual or potential value and, therefore, be capable of utilization. It should be recalled that utilization, as defined under Article 2(c) of the Nagoya Protocol, covers research and development exploiting the “genetic and/or biochemical composition of genetic resources including through the application of biotechnology.” It is tempting to confine “biotechnology,” as defined in that article, to technology applying tangible resources, rather than intangible information. However, the preceding phrase, “research and development on the genetic and/or biochemical composition of genetic resources” suggests that utilization of genetic resources can also occur through information repositories and dissemination of sequence information ([55], p. 9; [11], p. 8).

58Further, as already noted, it is arguable from the Nagoya Protocol’s definition of “derivative,” that a genetic material remains valuable, even without functional units of heredity. That instrument also clarifies that utilization of genetic resources can be through biotechnology. In other words, utilization covers the application of biotechnology, and the latter includes any technological application that uses the derivatives of biological systems or living organisms to develop or improve useful products or processes. In this context, experts suggest that derivatives which, according to the Nagoya Protocol, do not need to have functional units of heredity, can also include information derived from genetic resources ([45], p. 9; [47], p. 65).

It means that, as defined in the CBD, biotechnology is more expansive than genetic engineering and covers synthetic biology ([47], p. 67). This extends the Nagoya Protocol’s coverage to technologies applying material that lacks functional units of heredity ([47], p. 67). What follows, on the whole, is that, as highlighted earlier under the section of this article explaining the importance of digital sequence information, synthetic biology involves the utilization of genetic resources, including digital sequence information. These resources provide raw material for biotechnological research ([76], p. 161).^59^ Barring human or machined-designed sequences, the exploitation of digital sequence information is usually made possible through initial access to the regular genetic resource from which it originated ([10], p. 4).

Reinforcing the above view, Muller observes that biotechnological research is directed at the informational element of genetic resources ([65], pp. 14–15). He explains that, although the CBD and the Nagoya Protocol incorrectly focus on the physical enclosure, in terms of biotechnological research, it is in the intangible informational content that value really lies. Thus, genetic resources and the object of “access” under the ABS system should more appropriately be understood as “natural information.” Since the natural informational content, not the physical material, holds the potential value for biotechnological research, it should be the focus of attention ([65], p. 15). It is this informational element, combined with human ingenuity, in the form of biotechnology, which propels research and development, resulting in biotechnological products and services ([65], p. 16).

Likewise, Lawson observes that, while international ABS regimes, such as the CBD and the Nagoya Protocol, as well as the ITPGRFA^60^ and WHO’s International Health Regulations^61^ focus on access to and benefit-sharing from the tangible material constituting genetic resources, a key component is the information borne by such material ([77], p. 2). In effect, genetic resources are simply “packets of informational goods” existing in a physical biological form ([51], p. 14). It is this informational content, rather than the physical material, that provides value for biotechnological research.

With the advent of bioinformatics, such information can be extracted and deposited in databases from where they can openly be accessed and utilized without contact with physical samples. Thus, diverse ways of utilizing genetic resources have emerged that include sundry forms of sequence data held in globally accessible public databases for assorted uses [51],^62^ p. 25; [18], pp. 8, 27). Even WHO now acknowledges that it is possible to synthesize vaccine viruses simply by relying on digital sequence information ([78], pp. 5, 8). Hence, the utilization of digital sequence information derived from regular genetic resources that excludes benefit-sharing could be viewed as the “digital misappropriation” of genetic resources [3] pp. 1, 10]. To focus solely on physical and full-coding genetic material would undermine the CBD. It might then be asked whether the utilization of digital sequence information, coding or non-coding, actually amounts to the utilization of genetic resources. This is unclear from the CBD, which hardly envisaged this type of resource when it was negotiated. It will, nevertheless, be argued that utilization, as defined under the Nagoya Protocol, covers all uses of genetic resources, whether accessed physically or digitally. Unlike most biological resources, genetic resources, although physical in nature, can be converted into a non-physical, informational form, with their original value intact ([79], p. 1). Value is not eroded simply because they are stored and accessed in a digital form. Indeed, without value, there would be no desire to access them. The foregoing arguments are upheld by expert reports and domestic ABS laws.

### Expert Reports

The Report of the Meeting of the [CBD’s] Group of Technical and Legal Experts on Concepts, Terms, Working Definitions and Sectoral Approaches ([45], p. 8) provides representative forms of utilization capable of creating ABS obligations. Relevant for the present purposes are such uses as gene sequencing, the creation of reference collections, and synthesis of DNA segments.^63^ There are also suggestions that, while the CBD and the Nagoya Protocol were aimed primarily at tangible genetic material, sometime in 2008, during negotiations over the Nagoya Protocol, collection of data on genetic resources was subtly incorporated into the ABS system through the definition of “derivative”; a term considered to cover sequence information obtained from genetic material ([55], p. 10).

Additionally, a report by the Organization for Economic Cooperation and Development (OECD) suggests that “utilization” of genetic resources includes bioinformatics. This covers the construction of databases on genomes and protein sequences, as well as the modeling of complex biological processes, including through the use of systems biology [80]. The OECD’s categorization implies that the utilization of genetic resources that would trigger ABS obligations covers bioinformatics. This means the digital management of biological information, and encompasses virtual libraries or databases for genetic sequences ([37], p. 39) full-coding or otherwise. Similarly, the more recent CBD-commissioned Study 1 observes that bioinformatics, sequence databases, registries of standard biological parts and gene synthesis services rely on digital sequence information, which is capable of attracting ABS obligations. It acknowledges that digital sequence information can result from the utilization of genetic material, rendering it subject to benefit-sharing obligations ([17], p. 29). The Study, however, queries whether ABS conditions should apply to the utilization of digital sequence information per se, such as when obtained from a database, such as INSDC ([17], p. 29).

In the above regard, the EU’s position is that, until international agreement is reached on the issue by Parties to the Nagoya Protocol, the utilization of digital sequence information obtained from public databases is not covered by the Nagoya Protocol and related EU regulations,^64^ in the absence of access to or utilization of the genetic material from which the digital sequence information originated ([81], p. 10).^65^ It means that users within the Union would not be subject to ABS obligations in such cases. This position, which is comparable to that of the Australian Commonwealth, is however, contestable. Arguably, as long as the implicated digital sequence information is traceable to a regular genetic resource, ABS obligations ought to apply; otherwise, the ABS system would become porous.^66^

The above arguments are consistent with other authoritative commentaries. In a report written by the Fridtjof Nansen Institute for the CBD, Schei and Tvedt maintain that bioinformatics provides the means for “realizing the value in the genetic material disconnected from the biological sources where it was originally found” ([68], p. 15). Through such means, significant amounts of genetic sequence information are collected in non-commercial research projects, digitized, and stored in freely accessible online databases ([8], p. 8). Hence, the authors argue that, if digital sequence information is excluded from the meaning of genetic resources, “valuable ways of realizing the potential value of the functional units of heredity could fall outside the scope of an international access and benefit sharing regime” ([68], p. 25). Such apprehension is justified. Accentuation of tangibility and functional units of heredity obscures the intricacy of genetic resources, their informational nature, and importance for the development of novel biotechnologies. Current synthetic biology and related research increasingly relies on digital sequence information, instead of actual molecules. Lawson rightly observes that, with the advent of computer technologies, attention is shifting from physical genetic material to its non-material value ([77], p. 3). This “dematerialization” of genetic resources suggests an “increasing trend for the information and knowledge content of genetic material to be extracted, processed, and exchanged in its own right, detached from the physical exchange of the plant genetic material” ([77], p. 3 [82];).

Although synthetic biology remains largely a “wet laboratory science” ([83], pp. 7, 11) digital sequence information is fast becoming the new “raw material” that synthetic biologists use in creating or modifying living systems ([84], p. 26). Instead of scouting for natural genes, they can now download digital sequence information from publicly accessible databases and have it synthesized by gene services companies for new product innovation. Therefore, exclusion of digital sequence information from the meaning of genetic resources due to intangibility would enfeeble the ABS system. It would mean that even where sequences have been extracted from physical genetic resources, transferred, and stored digitally through bioinformatics for subsequent utilization, without the knowledge and consent of their owners, they would not, as the EU and other jurisdictions suggest, be covered by the ABS system. With growing levels of sequence information being freely disseminated through online databases, coupled with increasing efficiency and affordability of gene synthesis services, concerns over the digital misappropriation of the genetic resources of provider states would likely increase ([85], p. 6).

Avoiding such an anomaly requires an approach that considers research and development activities applying digital sequence information as the utilization of genetic resources, consistent with Nagoya Protocol provisions. Insofar as synthetic biology research, especially the top-down approach, involves the utilization of digital sequence information derived from regular genetic resources, the ABS system ought to apply. To fully achieve CBD objectives, providers of genetic resources should share in the benefits arising from the commercial and other exploitation of their resources, whether constituted in tangible or intangible form ([51], p. 7). Article 15(7) of the CBD clearly requires the fair and equitable sharing of results from research and development, as well as benefits obtained from the commercialization and “other utilization of genetic resources.”

Similarly, Article 5(1) of the Nagoya Protocol requires the fair and equitable sharing of benefits from the utilization of genetic resources, including “subsequent applications and commercialization.” This quotation has a potentially wide coverage that could also include uses of non-naturally occurring components, such as digital sequence information, given the Protocol’s definition of “utilization of genetic resources,” “derivatives,” and “biotechnology” ([47], pp. 69–70; [55], p. 9). Such dynamism is more suitable for the interpretation of the CBD and full realization of its ABS objective.

### Domestic Access and Benefit-Sharing Laws

Although the framing of the CBD and the Nagoya Protocol suggests that intangible sequence information falls outside the international ABS regime, since the Nagoya Protocol only provides minimum implementation standards, parties can determine what would attract ABS obligations in their domestic ABS or related measures ([8], p. 13; [11], p. 11). Expectedly, some states have adopted measures expressly including digital sequence information or simply given expansive interpretation to current measures so as to cover such information ([11], pp. 11–13). For example, some domestic and regional laws implementing the international ABS regime define genetic resources to cover digital sequence information. This can be seen in the ABS regime^67^ of the Andean Community comprising Bolivia, Colombia, Ecuador, and Peru, which provides that genetic resources cover “all biological material that contains genetic information of value or of real or potential use.” Nevertheless, this provision arguably refers, not to genetic information per se, but “biological material”; the tangible container of that information. 

Muller rightly charges that provision with “double think” ([65], p. 18). Although purporting to define genetic resources as information, an intangible, it conceives such information in material terms. By focusing on the physical receptacle of genetic information, instead of the information itself, it wrongly regulates the former rather than the latter, as the object of access ([65], p. 18). However, that law potentially covers genetic information by also providing that “access” is “the obtaining and use of genetic resources conserved in situ and ex situ, of their byproducts and, if applicable, of their “intangible components,” for purposes of research, biological prospecting, conservation, industrial application and commercial use, among other things.”^68^ Based on the above provision, in Colombia, the use of digital sequence information obtained directly from a physical genetic resource or a database for the purposes mentioned above is subject to an ABS agreement ([11], p. 15). The draft ABS regulation of Peru, another Andean Community member, specifically covers genetic information, which is defined as “a sequence of nucleotides, including digitally stored sequences” ([11], p. 16)^69^ Brazil’s former ABS law^70^ similarly exhibited the problem of “double think” by providing that “genetic heritage” is “information of genetic origin contained in samples of all or part of a plant, fungal, microbial or animal species in the form of molecules and substances.”

That phrase, “in the form of molecules and substances,” suggests that the object of regulation is tangible material, not intangible information, as ostensibly intended. The country’s new ABS legislation^71^ more specifically refers to genetic information. It applies to “information of genetic origin from plants, animals, microorganisms or species of other kinds, including substances derived from the metabolism of these living beings” ([86], p. 1).^72^ Importantly, the law clarifies that it applies to the utilization of sequence information posted in a public database, such as GenBank,^73^ and presumably INSDC, contrary to the position taken by the EU and others.

In India, ABS measures include the Biological Diversity Act (BDA) and the Access and Benefit-Sharing Guidelines, both of which also allude to digital sequence information derived through access to biological resources, warranting benefit-sharing [11], p. 53).^74^ Similarly, Malaysia’s ABS law expressly provides that “biological resources” also include information relating to “genetic resources, organisms, microorganisms, derivatives and parts of the genetic resources, organisms, microorganisms or derivatives.”^75^ As well, China maintains a definition of “biological genetic resources” that also covers information and data derived from “materials and derivatives containing biological genetic functions” ([11], p. 14). In South Africa, the definition of genetic resources covers “genetic potential, characteristics or information of any species.”^76^ The reference to “genetic potential, characteristics or information” brings digital sequence information within the meaning of genetic resources, uses of which attract benefit-sharing obligations ([11], pp. 17–18). Likewise, the ABS system in Kenya regulates digital sequence information by defining “access” as “obtaining, possessing and using genetic resources conserved, whether derived products and, where applicable, intangible components, for purposes of research, bioprospecting, conservation, industrial application or commercial use.”^77^ Here, “intangible components” covers information relating to the country’s genetic resources ([11], p. 15).

Nevertheless, some countries have expressly excluded digital sequence information from their domestic ABS regimes. For example, Japan’s ABS guidelines do not consider digital sequence information as falling within the meaning of genetic resources, and expressly exclude “information concerning genetic resources, such as nucleic acid base sequences.”^78^ As such, prior informed consent is not required to access digital sequence information or even physical genetic resources ([11], pp. 20, 28). Likewise, the Commonwealth of Australia distinguishes digital sequence from tangible genetic resources, maintaining that the former lacks functional units of heredity and falls outside the CBD, as well as the Nagoya Protocol ([11], p. 38). By contrast, the ABS system of the Queensland territory of Australia covers genetic information ([11], pp. 38–39).^79^ The territory’s Biodiscovery Act is activated when native biological resources are taken and used for biodiscovery research,^80^ which is defined as “the analysis of molecular, biochemical or genetic information about native biological material for the purpose of commercializing the material.”^81^ The territory’s model ABS clauses also cover digital sequence information ([11], p. 39).

Notably though, in both the Commonwealth and the Queensland territory of Australia, digital sequence information accessed from a database and used independently of a physical genetic resource to develop and commercialize a product, is not covered, unless expressly agreed at the time of access to the physical genetic resource ([11], p. 40). In fact, in both jurisdictions, digital sequence information does not qualify as a genetic resource in the language of the CBD, although in the Queensland territory, it may be seen to arise from the utilization of a physical genetic resource, consequently warranting benefit-sharing obligations ([11], p. 40).^82^

It is evident from the foregoing review that, although some countries have expressly excluded digital sequence information from their ABS systems, several others have either expressly included it within the meaning of genetic resources in their ABS systems or adopted a broad interpretation of genetic resources in existing ABS regimes so as to cover it. Thus, access to digital sequence information is equated with access to a physical genetic resource, which imposes benefit-sharing obligations, given that the former is derived from the utilization of the latter. *In Association for Molecular Pathology *et al*. v Myriad Genetics *et al.,^83^ both the District Court and the U.S. Supreme Court observed that genetic information is what makes a genetic material valuable for biotechnological research. Generally, harnessing such information involves initial access to the originating genetic resource ([10], p. 4). Understandably, provider states consider it a genetic resource.

Overall, it can be concluded that digital sequence information, as “the bioinformatic decoding” of physical genetic resources clearly amounts to the utilization of such resources. Indeed, since genome sequencing is an indispensable enabler of synthetic biology, the latter can be seen as a benefit arising from the former. Consequently, the synthetic biology field, as a benefit, is appropriately subject to the CBD’s objective of ensuring the fair and equitable sharing of benefits arising from the utilization of genetic resources through sequencing processes. This provides a sound rationale to extend the reach of the CBD and the Nagoya Protocol to the use of digital sequence information as a key resource in the synthetic biology field.

In the above regard, it is vital to recall that Decision XIII/17^84^ adopted at COP-13 notes that AHTEG on Synthetic Biology advances an operational definition of the field as “a further development and new dimension of modern biotechnology that combines science, technology and engineering to facilitate and accelerate the understanding, design, redesign, manufacture and/or modification of genetic materials, living organisms and biological systems,” which is considered as a useful starting point for scientific and technical discussions under the CBD and its Protocols ([87], p. 1).^85^ That synthetic biology is now expressly considered as “a further development and new dimension of modern biotechnology,” reinforces the nexus between this field and the original language and objectives of the CBD. This evolutionary approach is necessary to align the CBD with scientific progress and ensure its continuing relevance. The following analysis of treaty, as well as case law, further illuminates this matter.

### Treaty Interpretation and Case Law

Scholarship on the applicability of the ABS regime to digital sequence information mostly engages with the CBD, the Nagoya Protocol, and domestic ABS provisions. However, as a further contribution to the scholarly heritage, this article incorporates treaty interpretation and case law. Doing so significantly guides the interpretation of the contested terms, “genetic resources” and “functional units of heredity.” As treaties, the CBD and the Nagoya Protocol come within the realm of public international law. A pertinent question is how they should be interpreted amid uncertainty. The CBD provides that disputes over its interpretation or application shall be resolved through negotiation,^86^ conciliation,^87^ or arbitration.^88^ These provisions also apply to disputes over the interpretation or application of the Nagoya Protocol.

Despite their acknowledged merits, those alternative dispute resolution methods have limited value for the present purposes. Generally, the outcomes of negotiation or conciliation proceedings do not bind the parties concerned. Arbitral awards are binding, but only on the parties to the arbitration proceedings. Hence, they have limited precedential value. Consequently, arbitral tribunals presiding over disputes with substantially similar facts and legal questions may make directly opposite awards.^89^ Nevertheless, Article 4, Annex II, Part 1 of the CBD provides that arbitration proceedings shall be in accordance with the provisions of the Convention, any relevant protocols and, importantly, international law. According to Article 27(3)(b) thereof, disputes over the interpretation or application of the instrument can also be settled by the International Court of Justice (ICJ).

Under international law, treaty interpretation is governed by customary international law as the established practices among states; the Vienna Convention; judicial decisions; and the teachings of the most highly qualified publicists.^90^ Articles 31–33 of the Vienna Convention state the general principles of interpretation. Under Article 31(1), “a treaty shall be interpreted in good faith in accordance with the ordinary meaning to be given to the terms of the treaty in their context and in the light of its object and purpose.”^91^ While a treaty provision is to be given its ordinary meaning, based on Article 31(1), such meaning may change over time. The Vienna Convention does not tell whether the ordinary meaning of a provision, as understood at the time of its adoption, or at the time of its interpretation, should apply ([58], p. 131).

Yet, to remain relevant, laws must metamorphose with changes in the times and circumstances of their adoption ([88], p. 365), although adaptability, itself, must be integrated with the imperative of legal certainty ([89], p. 4). A key device for ensuring such adaptability is evolutionary interpretation. This requires a treaty term to be interpreted in a dynamic manner in line with changing situations. Evolutionary interpretation assumes that parties may intend a treaty or some of its provisions to evolve in meaning with “changes in factual or legal circumstances ranging from scientific or technical developments to the emergence of new legal regimes” ([90], p. 18).

Evolutionary interpretation of a treaty term may also be justified by the requirement in Article 31 of the Vienna Convention to interpret a treaty in good faith, consistent with its object and purpose, taking into account relevant international law rules ([63], pp. 8, 14–16).^92^ Publicists also acknowledge that the terms, objects, and purposes, as well as intentions of a treaty may justify evolutionary interpretation ([58], pp. 168–169). Moreover, ICJ opinions, which encapsulate customary international law, support evolutionary interpretation, where the terms of a treaty imply that the text may admit of “factual or legal evolution after its conclusion.”

In *Namibia Legal Consequences*,^93^ for example, the ICJ stated that where the terms of a treaty are “by definition evolutionary,” their “interpretation cannot remain unaffected by the subsequent development of law.”^94^ Hence, the Court concluded that the terms, “strenuous conditions of the modern world,” “‘the wellbeing and development’ of the peoples concerned,” and “sacred trust” used in the Covenant of the League of Nations,^95^ were evolutionary, not static.^96^ Again, in *AegeanSea*,^97^ the Court held that the term, “rights” was “generic” and must, consequently, “evolve in meaning” consistent with “the development of international relations.”^98^ In *Gabcikovo-Nagymaros Project*,^99^ the Court also stressed that a 1977 Hungary-Slovakia treaty for the construction of barrages on the River Danube, traversing both countries, was not static, but “evolving” and, therefore, amenable to newly emerged peremptory norms of general international law. Thus, “current standards” of environmental protection were relevant to its implementation.^100^

In giving evolutionary interpretations to the treaties involved in the foregoing three cases, the ICJ considered the parties’ intention. They were presumed to have used the relevant treaty terms intending them to evolve with time ([90], p. 20). In *Namibia Legal Consequences*, for example, the Court first alluded to “the primary necessity of interpreting an instrument according to the intention of the parties at the time of its conclusion.”^101^ It then proceeded to hold that they “must be deemed to have accepted” that the treaty should evolve,^102^ unless there was unmistakable evidence suggesting otherwise. Therefore, the Court concluded that South Africa’s obligations toward the Namibian people under the “sacred trust” had been affected by changes occurring after the Covenant of the League of Nations was made.^103^ The Court continued that “the objective of the sacred trust was the self-determination and independence of the peoples concerned,” even though such a right was probably not contemplated when the Covenant was concluded.

In *Dispute Regarding Navigational and Related Rights*,^104^ the ICJ crystallized the above principles into a “general rule” for determining when parties’ intention must be presumed to be evolutionary.^105^ According to the Court, when generic terms are used in a treaty and that treaty is meant to have a “continuing duration,” the parties “must” be deemed to have intended such terms to be given an evolutionary interpretation. Impliedly, a term would most likely be considered evolutionary because it is generic and there is further “indication that the term was supposed to connote a perpetual obligation whose meaning would have to be keyed to changing factual or legal circumstances to remain relevant as a right over time” ([90], p. 22). Thus, in the above case, the Court considered the treaty term, “comercio,” to be generic and the 1858 Costa Rica-Nicaragua treaty to have been intended to have a perpetual duration.^106^ On both grounds, it concluded that the parties intended “comercio” to evolve so as to include activities that were not viewed as “comercio” when the treaty was adopted. Instead of limiting the term to its original 1858 meaning of trade in goods, the Court adopted a broad interpretation covering trade in goods and services, including tourism.^107^

In *Aegean Sea*, the ICJ similarly ruled that the generic term, “territorial status,” used in an instrument intended to have a “continuing duration,”^108^ must be presumed to have been intended to “follow the evolution of the law.” The International Law Commission’s *Conclusions on the Fragmentation of International Law* clarifies that, based on Article 31(3)(c) of the Vienna Convention, this new meaning of a generic term shall be determined from the international law applicable in relations between the parties at the time of the treaty’s application ([63], pp. 15–17). In effect, generic terms are to be interpreted based on their international law meaning at the time of their application.^109^

Parties’ intention need not be explicit ([90], p. 20). As the ICJ stated in *Dispute Regarding Navigational and Related Rights*, it may be presumed.^110^ According to the Court, “there are situations in which the parties’ intent upon conclusion of the treaty was, *or may be presumed to have been*, to give the terms used—or some of them—a meaning or content capable of evolving, not one fixed once and for all.”^111^ In the above case*,* the ICJ noted that “generic terms” “[refer] to a class of [something].”^112^ They mean an indefinite category of referents that “share some particular trait(s)” ([58], pp. 177–178; [91], pp. 178, 194). It would be recalled that, in *Aegean Sea,* as well as *Dispute Regarding Navigational and Related Rights*, the terms, “territorial status” and “comercio,” were respectively considered as generic. In both cases, the referents were indefinite; the former, “a class of issues” and the latter, “a class of activity” ([58], p. 178).^113^ Since both terms had evolved, they were given an evolutionary interpretation based on the ICJ’s general rule. Generic terms may be viewed as vague in that the reference may remain the same, while its scope may vary with changing times: this makes it reasonable to infer that a text was intended to evolve with the changing scope of the reference, hence justifying evolutionary interpretation ([58], p. 181). Thus, in *Dispute Regarding Navigational and Related Rights*, the reference, “comercio,” was vague, and the ICJ resolved this vagueness by giving that term an evolutionary interpretation. That helped to align it with changes that had occurred in its scope ([58], p. 182).

Decisions of quasi-judicial bodies similarly reflect evolutionary treaty interpretation. In *United States-Import Prohibition of Certain Shrimp and Shrimp Products*,^114^ the Appellate Body of the World Trade Organization (WTO) ruled that “exhaustible resources’ used in exception XX(g) of the General Agreement on Tariffs and Trade (GATT) 1994, covered not only non-living resources, such as minerals, which are finite, but also living or biological resources, such as sea turtles, which are renewable.^115^ This is because, although reproducible and therefore renewable, living species could be depleted and endangered by human activities.

Instructively, the Appellate Body emphasized that, although the GATT language, “resources,” was used over 50 years ago and subsequently transplanted in an unmodified form into the WTO Agreements in 1994, that term was not static in reference, but “by definition evolutionary.”^116^ It, therefore, warranted evolutionary interpretation in line with international concerns for environmental protection and conservation, which had subsequently emerged since that time. The Appellate Body thus resolved the vagueness of the contested term, “exhaustible resources,” with the aid of evolutionary interpretation, which considered changes that had since occurred in its scope. In doing so, the Appellate Body tellingly drew upon other international instruments, particularly the CBD, which uses terms, such as “biological resources” and “marine living resources.”

In interpreting “exhaustible resources” to include living and non−living resources, the Appellate Body also referred to* Namibia legal Consequences*, as well as the United Nations Convention on the Law of the Sea (UNCLOS) 1992,^117^ the latter of which refers to both “living” and “non−living” resources. It observed that the UNCLOS provisions on fisheries largely reflected customary international law. Deploying Article 31(3)(c) of the Vienna Convention, the Appellate Body stressed that “our task here is to interpret the language of the chapeau [of Article XX of GATT], seeking interpretive guidance as appropriate, from the general principles of international law.”^118^

In its *Conclusions on the Fragmentation of International Law*, the International Law Commission similarly maintains that a treaty term may be presumed to have been intended to have an evolutionary meaning where it requires taking into account subsequent changes of technical, legal, or economic nature or where that term is of such a general character as to necessitate taking into consideration changing situations ([63], p. 16). It referred to the ICJ’s remark in *Gabcikovo-NagymarosProject* that the parties’ use of technical terms in the treaty at issue showed that they “recognized the potential necessity to adapt the Project.” Consequently, the treaty was not static.^119^ In *United States-Import Prohibition of Certain Shrimp and Shrimp Products* where the parties used the generic term, “exhaustible natural resources,” the Appellate Body followed the same approach when it stated that, “we note that the generic term ‘natural resources’ in Article XX(g) is not ‘static’ in its content or reference, but is rather ‘by definition evolutionary.’”^120^ Thus, where parties have used a scientific term or one expressed in such a general way, it may be concluded that they “did not intend to fix its meaning and thereby potentially ground their future obligations on outmoded or falsified scientific concepts, but intended the terms to connote obligations keyed to the evolving meaning of their terms” ([90], p. 21).

Altogether, the real essence of evolutionary interpretation is to ensure that a treaty effectively achieves its object and purpose, having regard to changes in its circumstances. Thus, in Iron Rhine Arbitration,^121^ even though the dispute between the parties did not concern generic terms, but technical problems over the rail route connecting them, the Permanent Court of Arbitration insisted that, “but here too, it seems that an evolutive interpretation which would ensure an application of the treaty that would be effective in terms of its object and purpose, will be preferred to a strict application of the intertemporal rule.”^122^ Reiterating the principle of effectiveness articulated in Iron Rhine Arbitration, the European Court of Human Rights similarly stressed the importance of interpreting the European Convention on Human Rights “in a manner which renders its rights practical and effective, not theoretical and illusory. A failure by the Court to maintain a dynamic and evolutive approach would risk rendering it a bar to reform and improvement.”^123^

From the foregoing analysis, the following conclusions can be drawn with regard to the CBD. “Genetic resources” and “functional units of heredity” are scientific terms. Also, they are generic in that they merely refer to an indefinite category of referents. Further, they are vague because, as argued earlier, despite the references remaining the same, there is potential for their scope to evolve with changing times. Based on ICJ’s jurisprudence, such terms must, by definition, be deemed evolutionary. Even if this contention fails to hold, those terms would still warrant evolutionary interpretation based on the ICJ’s general rule. Not only are they generic, but also, the relevant treaties in which they are used, the CBD and the Nagoya Protocol, have continuing durations. It is, therefore, reasonable to presume an intention by the parties thereto that both texts should evolve with those changing references, thus justifying evolutionary interpretation of both terms.

Digital sequence information was most likely not contemplated when the CBD was adopted in 1992. However, since that time, profound changes have occurred in the life sciences, stirred by rapid technological advances across diverse fields. As a result, genetic resources now regularly assume an informational form, including genome sequences stored in public online databases, rendering them far more ubiquitous and accessible to the global public ([51], p. 26). Consequently, in line with the jurisprudence already analyzed, the vague term, “genetic resources,” must be interpreted in an evolutionary manner to reflect developments that have occurred since 1992. Equally important, the Appellate Body’s ruling in *United States-Import Prohibition of Certain Shrimp and Shrimp Products* shows that the term, “resources,” applies not only to living, but also non-living things, such as minerals and, analogously, digital sequence information ([92], p. 106).^124^

Despite controversies over whether a treaty may have only one or several objects and purposes ([93], pp. 5–7), three objectives can be identified for the CBD. According to Article 1 thereof, these “are the conservation of biological diversity, the sustainable use of its components, and the fair and equitable sharing of the benefits arising out of the utilization of genetic resources, including by appropriate access to genetic resources.” These complementary goals may be read conjointly to mean that the CBD’s overall object and purpose is the establishment of a system of unimpeded access to genetic resources, while also ensuring their sustainable use, as well as fair and equitable flow of benefits to providers.

Preamble 12 of the CBD recognizes the dependence of indigenous and local communities on such benefits. This benefit-sharing component of the treaty’s object and purpose is the reward that provider states expect from the treaty to compensate them as custodians of biodiversity and support their conservation efforts ([10], p. 6). Failure to achieve it would jeopardize the durability of this system of access to and conservation of biodiversity. Erosion of benefit-sharing would discourage provider states from concluding ABS agreements, even for non-commercial research of potential value to biodiversity conservation, which is also connected to the achievement of the UN’s 2030 Agenda for Sustainable Development ([24], pp. 2, 4). Provider states may become concerned that their resources would eventually be sequenced and distributed for commercial use, without any benefits accruing to them ([8], p. 15). Since the 1990s, there has been an expanding list of genome sequencing projects providing sequence information on diverse genetic resources ([51], p. 33; [94], p. D475). By 2019, there were more than 1600 biological databases ([18], pp. 2, 17).^125^ Most of the standard sequences are lodged with the leading nucleotide sequence database, the INSDC, which comprises the DNA Data Bank of Japan, the European Bioinformatics Institute [EMBL-EBI], and the U.S. National Center for Biotechnology Information that hosts the GenBank ([18], pp. 2, 24). By April 2019, GenBank, which has over 5.8 million users across the world, had over 212 million sequence data entries, 76% of them from plants, animals, bacteria, fungi, and viruses of relevance to Article 15 of the CBD ([18], pp. 28–29, 31). The EMBL-EBI, itself, records over 38 million requests every week ([24], p. 6).

As a whole, the INSDC has users from every country in the world. Most of them are, however, from developed countries, although Brazil, India, and Singapore are also emerging ([18], p. 31).^126^ Along with its three partner institutes and many other databases that access sequence data from its databases, the INSDC maintains a policy of open, unrestricted access to every user across the world, who has Internet connectivity ([18], pp. 26–27). Without login or registration requirements, users, including firms and research institutes, access INSDC entries “hundreds of thousands of times a day” ([18], p. 25). Together with its open access and no use restriction policy, the INSDC does not require depositors to furnish ABS-related information, such as proof of prior informed consent and mutually agreed terms ([95], p. 5; [24], pp. 5, 6).^127^ Users can obtain sequence data without ABS obligations or any means of tracing their origin, absent accession numbers. Moreover, even though accession numbers may enable traceability, this is not always fail-safe, once data leaves INSDC databases ([8], p. 11; [18], pp. 3, 35). In any case, INSDC-generated accession numbers are essentially meant to foster scientific publication, rather than serve regulatory purposes.

The INSDC databases contain a metadata tag for “/country,” which permits depositors to report the country of origin of nucleotide sequence data, but, as Study 2/3 observes, this term does not perfectly correspond to “country of origin.” Even at that, reporting of country of origin of INSDC entries remains poor. Despite a gradual increase in reporting, in 2018, barely over 40% of nucleotide sequence data deposited indicated a country of origin, which suggests that potentially thousands to millions of sequences do not carry country of origin information ([18], pp. 4, 65). Further, in jurisdictions, such as India and South Africa, country of origin is required for patent applications, yet such information is not disclosed when nucleotide sequence data related to patents is deposited with the INSDC ([18], p. 5).

While it may be possible to trace sequence data to the underlying genetic resource and country of origin, this is only if a depositor reports it ([18], p. 4). Depositors may, by error or willful intent, omit or enter incorrect country of origin. There may, in fact, be cases of fraudulent reporting. A maleficent INSDC user could develop a lucrative product by using “an NSD entry from a CBD Party and, although aware of ABS obligations, country of origin and, conceivably even access permits, in the process of patenting, lie about the AN [accession number] associated with this piece of NSD or lie about the presence of ABS obligations” ([18], p. 56). Likewise, other information that could provide legal certainty for both providers and users, such as “date of sampling/primary access” and “date of sequencing/utilization” do not always accompany sequence data submissions ([18], pp. 46, 65).

Surely, open access to digital sequence information is supportable and consistent with CBD requirements, particularly with regard to biodiversity conservation ([96], pp. 3–4; [97], p. 1). Open access obviates the need for physical access to genetic material and facilitates reproducibility, validation, and improvements on results, as well as research collaboration in a field like taxonomy that demands collectivism ([3], p. 26; [24], p. 6). Equally important, however, are the conditions for such access. Allowing access to information on the biodiversity of provider states, without benefit-sharing obligations, is difficult to justify. After all, as some have argued, genome sequencing projects may eventually charge rents, whether described as maintenance costs or otherwise, for access to their databases by parties ([95], p. 5)^128^ who also could use accessed sequences to develop profitable inventions and patent them ([24], p. 17).

It is reported, for example, that strain C15 of the Ebola virus, isolated from a sample obtained from Guinea, in West Africa, was sequenced and deposited in GenBank by European researchers. From there, it was accessed by U.S. firm, Regeneron Pharmaceuticals, which used it to develop an anti-Ebola drug that it patented in Nigeria and South Africa, two of the largest markets on the African continent ([86, p. 17; 105, pp. 1–3]. The costs of such products are usually unaffordable in many states that are ironically the providers of the underlying genetic resources. During the Indonesian bird flu (H5N1) crisis, for example, Indonesian strain of the virus shared by the country with WHO’s Global Influenza Surveillance Network, was accessed and used by foreign pharmaceutical firms to develop vaccines. Those vaccines were patented and sold at prices beyond the reach of afflicted Indonesians, leaving scores of them dead ([98, 99]).

States like Japan and Costa Rica view open access to digital sequence information derived from physical genetic resources as a non-monetary form of benefit-sharing; they support the deposit of sequence data in openly accessible databases to promote scientific development ([11], p. 27). This arrangement is, however, more likely to benefit states that have the necessary technological, human, and organizational capacities to exploit accessed data ([24], p. 6; [83], p. 10). The mostly developed user states are far better equipped in these areas than many provider states in the developing world. For example, most file transfer protocol downloads from the INSDC are by developed states, ostensibly because they have the facilities required for such purposes ([18], p. 32).^129^

Additionally, the INSDC partner institution, GenBank, allows depositors to claim intellectual property rights, including patents, in all or aspects of deposited data ([18], p. 27), potentially placing restriction on use. Also, data deposited with the INSDC may be accessed by private databases and combined with their in-house generated data for research and development. Yet, access to private databases is generally restricted [18], p. 36). Mindful of those factors, states, such as Brazil and South Africa, insist that “there is no national benefit from such international work and it is very difficult to trace the benefits that are being generated without an internationally agreed standard” ([11], p. 27). They argue that, even if open access to digital sequence information were to benefit society, there should also be concrete monetary benefits. Otherwise, provider states may be discouraged from cooperating with non-commercial research projects or compelled to tighten access conditions. In that case, the goal of orderly access and biodiversity conservation critical to sustain present and future generations would be undermined.^130^ Scientists would be unable or reluctant to engage in otherwise beneficial research, thus stifling scientific progress ([8], pp. 17–18). During its thirteenth meeting in December 2016, COP similarly acknowledged that digital sequence information on genetic resources is a cross-cutting issue, which is relevant to the three objectives of the CBD [12], in addition to the 2030 Agenda for Sustainable Development.

It is worth recalling that, in establishing the international ABS regime, CBD parties sought to ensure fair and equitable sharing of benefits from the utilization of genetic resources so as to promote access to sustainable use and conservation of biodiversity. To effectively pursue these goals and fully achieve the CBD’s object and purpose, “genetic resources” and “functional units of heredity” should be construed in such a dynamic manner as would include digital sequence information ([100], p. 3), regardless of intangibility, and whether full-coding or not. Current synthetic biology research typically replicates and tinkers with natural sequences and processes, aided by CRISPR-Cas 9 and other tools, to produce useful biological functions ([8], p. 9).

It is untenable that the genetic composition of a genetic resource utilized in such a way, even if transformed through biotechnology, should be excluded from the ABS regime, simply because it was accessed digitally or is non-coding. To give “genetic resources” its original, restrictive meaning would rob the ABS regime of the potential value of genetic material utilized in synthetic biology or other future biotechnologies ([7], p. 22). As Schei and Tvedt conclude, and in conformity with the jurisprudence earlier examined, “since potential value and the level of knowledge regarding functionality in biology change, the wording of the legally binding definition suggests being dynamic in the sense that it captures the evolving knowledge and technological state of the art” ([68], p. 10). This approach is needed to ensure that provider states benefit fairly and equitably from the use of digital sequence information derived from their regular genetic resources ([101], p. 9).

## Conclusion

This article has examined the applicability of the CBD and its ABS regime to digital sequence information on genetic resources. The CBD’s original wording apparently conceives genetic resources in material terms, and some observers view “functional units of heredity” as full-coding sequences, even though undefined by the CBD and the Nagoya Protocol. Nevertheless, relying on expert reports, studies, the CBD, the Nagoya Protocol and domestic, as well as regional ABS legislation, this article has argued that rigidly interpreting the CBD to exclude digital sequence information, full-coding or not, would weaken the ABS regime.

To remain relevant and achieve its objectives, the CBD must evolve with scientific progress. As the sequencing of regular genetic resources and open dissemination of sequence information expands, the potential for inappropriate access to the genetic resources of provider states would likely increase. This makes it appropriate to adopt a dynamic interpretation of the CBD, which views digital sequence information, full-coding or not, in the same way as their physical manifestations. In reinforcing these arguments, this article invoked evolutionary interpretation as a veritable tool for aligning the CBD and the Nagoya Protocol with the changes that have followed their adoption.

Treaties cannot anticipate all future possibilities and are usually drafted in general terms. Apart from the present, they may eventually have to address completely different future situations. However, once adopted, they remain static, despite changes in the scientific landscape that influenced their drafting. Article 29 of the CBD permits treaty amendment, but forging international consensus to amend treaties and reconcile them with new realities is difficult. Yet, to remain relevant, they must evolve with changing circumstances. Evolutionary interpretation helps in meeting this challenge.

Ultimately though, formal clarification is needed on the precise meaning of digital sequence information, its scope, whether narrow, broad or intermediate, and particularly, whether it is covered by the ABS regime. As well, ideal methods of tracing the generation and utilization of digital sequence information, identifying beneficiaries, and sharing benefits need to be settled, as is the distinction between data and information, which could be vital in determining the coverage of the concept of digital sequence information. Public databases, particularly the INSDC, could strive to facilitate traceability through more diligent country of origin reporting for environment-related sequence data submissions. In this regard, their involvement in CBD deliberations would be salient. The tracking of Internet Protocol addresses and use of blockchain system to monitor uses of sequences could be explored, together with likely challenges. Also, capacity building for developing provider states and the possibility of establishing a Global Multilateral Benefit-Sharing Mechanism suggested under Article 10 of the Nagoya Protocol could be considered as a way of ensuring fairer and more equitable benefit-sharing.

It is expected that Part 2 of COP-15 would make progress on the above matters. This would enhance hopes for the realization of ABS objectives and provide certainty for users of genetic sequences as to the scope of potential ABS obligations facing them. For now, researchers, including those from a jurisdiction, such as the USA, which is not subject to the CBD and the Nagoya Protocol, should note that they may be bound by domestic or regional ABS laws. Violations may incur penal sanctions or denial of valuable intellectual property in jurisdictions where protection is sought. It is prudent to seek information and advice from relevant sources, including National Focal Points, Competent National Authorities and the Access and Benefit-sharing Clearing House. Further developments, particularly the outcomes of COP meetings, should be monitored closely.

## Footnotes

1 See, e.g., Sects. 6, 55, India Biological Diversity Act 2002; Sects. 6, 7, Access to Biological Resources and Benefit Sharing Act of Malaysia 2017.

2 Pursuant to Sect. 25, India Patents Act 1970 as amended by Patents (Amendment) Act 2005.

3 Pursuant to Sect. 30(3a)(b), South Africa Patents Act 1978 as amended by Patents (Amendment) Act 2005.

4 Paragraph 7.

5 Paragraphs 11.

6 Paragraphs 1–2.

7 Paragraph 9(a)(b).

8 Paragraph 11.

9 Paragraph 11(a).

10 Paragraph 11(b).

11 Paragraph 11(c).

12 Paragraph 11(d).

13 Paragraph 11(e).

14 Paragraphs 11(f)(i)–(iv), 12.

15 Paragraph 12.

16 These are actually two studies—databases (Study 2) and traceability (Study 3). Given the conceptual linkage between both topics, they were addressed as a combined study, as the title suggests. This combined study is tagged Study 2/3 in the CBD literature. See, e.g., Study 1, 40; Study 2/3, 15.

17 E.g., Colombia, Sudan, Senegal, and Togo.

18 Preamble 4.

19 Paragraph 2.

20 Paragraph 9.

21 Further noting that “non-coding DNA is also brought within the scope of DSI [digital sequence information]”.

22 Article 1.

23 Preamble 4 and article 4(a).

24 Article 15(1).

25 Article 15(5).

26 Article 15(4).

27 Article 15(7).

28 Articles 5–7.

29 Article 3.

30 Articles 15–16 and 18.

31 Article 15.

32 Article 2(2).

33 Article 2(10).

34 Article 2(9).

35 See also Article 2(2), CBD 1992.

36 See, e.g., No 181, Part 8A.03, Australia’s Environment Protection and Biodiversity Regulations 2000 (defining “access to biological resources” as “the taking of biological resources of native species for research and development on any genetic resources, or biochemical compounds, comprising or contained in the biological resources”).

37 Noting that, “biotechnology is heavily reliant on genetic resources for the discovery of new products and processes…Estimates indicate that 20–25% of [the pharmaceutical] market is derived from genetic resources, with nearly 2 out of 3 antibacterial agents deriving from genetic resources”.

38 Observing that “value may also be derived information, such as functional or regulatory pathways, structural polymers or biological functions of an organism that are encoded for by the genetic material, including metabolic products that have some practical applications”.

39 See, e.g., article 3(3), Regulation (EU) 511/2014 on Compliance Measures for Users from the Nagoya Protocol on Access to Genetic Resources and the Fair and Equitable Sharing of Benefits Arising from their Utilization in the Union 2014 (Regulation (EU) 511/2014) (defining access as “acquisition of genetic resources or of traditional knowledge associated with genetic resources in a Party to the Nagoya Protocol”).

40 For example, article 2(8), Brazilian Law 13.123 2015 defines access as “research or technological development conducted on sample of genetic patrimony”.

41 See, e.g., article 2(1), Regulation (EU) 511/2014 (“this Regulation applies to genetic resources over which States exercise sovereign rights and to traditional knowledge associated with genetic resources that are accessed after the entry into force of the Nagoya Protocol for the Union”). This interpretation has the effect of excluding significant amounts of genetic resources accessed before the Nagoya Protocol took effect on 12 October 2014 and deposited in ex situ collections in the EU, even if utilization occurs after that date.

42 See, e.g., article 28, Vienna Convention on the Law of Treaties 1969.

43 See also article 27, CBD 1992 referring to disputes over its “interpretation or application”.

44 See also India – Patent Protection for Pharmaceutical and Agricultural Chemical Products [1998] WT/DS50/AB/R, I DSR 9.

45 See also Industria Nacional de Alimentos SA v Indalsa Peru [2003] ICSID Case No ARB/03/4.

46 Article 1.

47 See article 33(1).

48 According to the International Law Commission, article 31(3)(c), Vienna Convention 1969 and the principle of systemic integration “call upon a dispute-settlement body to situate the rules that are being invoked in the context of other rules and principles that might have bearing upon a case.” “In this process, the more concrete or immediately available sources are read against each other and against the general law ‘in the background.’” The International Law Commission stresses that, although the result of such a step is unclear, a more important consideration, from the point of view of that article, is “the ‘system’ of international law as a whole”.

49 See, e.g., Sect. 63(3)(a)(b), Part IX, Access to Biological Resources and Benefit Sharing Act of Malaysia 2017 (providing that parties in possession of biological resources or associated traditional knowledge for which no ABS agreement had been concluded, shall upon the coming into force of this Act conclude such agreement with the provider, “if there is a new use of the biological resource or traditional knowledge associated with the biological resource; or there is development of a new product arising from the biological resource or traditional knowledge associated with the biological resource”).

50 See, e.g., article 14(1), Articles on Responsibility of States for Internationally Wrongful Acts, UN General Assembly Resolution 65/19, 6 December 2010 (distinguishing between acts and their effects).

51 This reasoning can be likened to the practice of a state, such as Brazil, which requires benefit-sharing, but not access, and has, for example, adopted a benefit-sharing measure for the utilization of digital sequence information independently of access to a physical genetic resource. See Study 4.

52 Article 14(3), Articles on Responsibility of States for Internationally Wrongful Acts, UN General Assembly Resolution 65/19, 6 December 2010 (imposing an obligation on states to prevent wrongful acts). See similarly, articles 15–16, 18, Nagoya Protocol 2010 (obliging states to ensure that genetic resources used in their territories comply with domestic ABS laws and to assist in redressing breaches).

53 See the comparable reasoning of the Appellate Body of the WTO in EC and Certain Member States—Large Civil Aircraft [2011] WT/DS316/AB/R 18, paragraph 683.

54 The Mavrommatis Concessions [1924] PCIJ Rep. Series B No 3, paragraph 86 (extending the effect of a Protocol to legal situations that preceded its coming into existence so as not to render it ineffective “with regard to the very period in which the rights in question are most in need of protection”). See more particularly, EC and Certain Member States—Large Civil Aircraft [2011] WT/DS316/AB/R 18, paragraphs 659, 662, 665-669, 671-672, 683, 689 (the Appellate Body of the WTO, citing the International Law Commission, adopted a similar approach by ruling that the non-retroactivity principle did not preclude application of Article 5 of the Subsidies and Countervailing Measures Agreement to the act of granting subsidies which occurred before the WTO Agreements came into force on 1 January 1995. This is because that act resulted in a situation causing adverse effects to the interests of other member states, which continued even after the Agreements took effect).

55 Article 32, Vienna Convention 1969 (in interpreting a treaty, resort could be had to supplementary sources, such as the preparatory work of the treaty and the circumstances of its conclusion). See also Medellin v Texas [2008] 28 USSC 1346, 1357 (U.S. Supreme Court observing, in relation to a treaty ratified by the USA, that it is also necessary, as aids to its interpretation, to consider the negotiation and drafting history of the treaty, including the common understanding of the parties thereto following its ratification).

56 Noting that, “the functions of many genes in even the simplest forms of life, like bacteria and yeast,” remain unknown.

57 According to one definition considered in Study 1, “NSD [nucleotide sequence data] include non-coding and coding sequences, regulatory sequences, conserved sequences, genes that encode specific traits, DNA without known function and ‘junk DNA’”.

58 The authors of CBD-commissioned Study 4; noting that they “have adopted a broad understanding of the word ‘use,’ which might potentially include the production, analysis, sharing, publication, and patenting of DSI, and other forms of commercial and non-commercial use”.

59 Observing that the development of products in the pharmaceutical, cosmetics, agricultural and other industries have traditionally relied on natural compounds, which continue to attract significant interest.

60 2001.

61 2005.

62 Envisaging a shift in which bioprospecting will no longer be dominated by biologists, but bioinformatists and computational biologists, who will continually mine growing levels of genomic and allied data in public and private repositories for every possible lead to produce desired functions.

63 Paragraph 13(6).

64 See, e.g., Regulation (EU) No 511/2014; Commission Implementing Regulation EU 2015/1866 laying down detailed rules for the implementation of Regulation (EU) 511/2014 of the European Parliament and of the Council as regards the register of collection, monitoring user compliance and best practices 2015; European Commission Guidance Document on the Scope of Application and Core Obligations of Regulation (EU) No 511/2014 of the European Parliament and of the Council on the Compliance Measures for Users from the Nagoya Protocol on Access to Genetic Resources and the Fair and Equitable Sharing of Benefits Arising from their Utilisation in the Union (EU Guidance Document), Commission Notice 2016/C 313/01, Official Journal of the European Union C 313, 27.8.2016.

65 2.3.3, EU Guidance Document.

66 The EU, however, recognizes that the utilization or publication of digital sequence information may be covered by conditions specified in mutually agreed terms by provider states and urges EU users to abide by such terms. See 2.3.3, EU Guidance Document (stating that the “use or publication of such data might be covered by conditions set in the mutually agreed terms, which should be respected. In particular, those who accessed the genetic resources and obtain sequence data from them should respect the conditions of the agreement entered into, and inform subsequent actors about any rights and obligations attached to the data obtained and related to any further uses of it”). Also, in respect of physical genetic resources and related traditional knowledge, article 4(1)(5), Regulation (EU) No 511/2014 require EU users to diligently ensure compliance with the ABS regulations of provider states, and where in doubt, seek authorization for access or abandon utilization altogether.

67 Article 1, Community Commission Decision 391 Establishing the Common Regime on Access to Genetic Resources 1996.

68 Article 1.

69 See also Ministerial Resolution 205–2019-MINAM.

70 Article 7(j), Provisional Act No 2.186–16 2001.

71 Brazil Law No 13.123 2015.

72 See Article 2.

73 Article 2(9). Nonetheless, there would be no benefit-sharing, if utilization does not yield something of economic value.

74 See Sects. 2(c)(d), 3, 4, Biological Diversity Act 2002; Regulation 1, Access and Benefit-Sharing Guidelines 2014.

75 Section 4, Part I, Access to Biological Resources and Benefit Sharing Act of Malaysia 2017.

76 Section 1(i), South Africa National Environmental Management: Biodiversity Act 2004 as amended by Act No 14 2013.

77 Part 1, Regulation 2, Environmental Management and Coordination (Conservation of Biological Diversity and Resources, Access to Genetic Resources and Benefit Sharing) Regulations 2006.

78 Chapter 1, article 3.1(1), Japan Guidelines on Access to Genetic Resources and the Fair and Equitable Sharing of Benefits Arising from Their Utilization 2017.

79 Sections 3, Biodiscovery Act 2004.

80 Section 5.

81 Section 5.

82 The territory’s law presently covers only digital sequence information derived from an accessed physical genetic resource for commercial purposes.

83 Association for Molecular Pathology et al. v Myriad Genetics et al. [2013] 569 US.

84 Paragraph 4.

85 Paragraph 4.

86 Article 27(1).

87 Article 27(4).

88 Article 27(3)(a).

89 See, e.g., SGS v Philippines [2003] ICSID Case No ARB/01/13 (tribunal ruling that breach of service contract also amounted to breach of bilateral investment treaty). C/f SGS v Pakistan [2004] ICSID Case No ARB/02/6 (another tribunal ruling that breach of service contract did not amount to breach of bilateral investment treaty).

90 Article 38(1)(d), Statute of the International Court of Justice 1945.

91 The context shall include annexes, preambles, as well as instruments and agreements made by the parties in connection with the treaty’s conclusion (Article 31(2)(a)(b)).

92 See also Iron Rhine (Ijzeren Rijn) Railway (Belgium v Netherlands) [2005] 27 R.I.A.A, paragraph 79.

93 Legal Consequences for States of the Continued Presence of South Africa in Namibia (South West Africa) Notwithstanding Security Council Resolution 276 (1970) [1971] ICJ Rep 16.

94 Paragraph 53.

95 1919.

96 Paragraph 53.

97 Aegean Sea Continental Shelf Case (Greece v Turkey) [1978] ICJ Rep 3.

98 Paragraph 78.

99 Case Concerning the Gabcikovo-Nagymaros Project (Hungary v Slovakia) [1997] ICJ Rep 7.

100 Paragraphs 122, 140.

101 Paragraph 53.

102 Paragraph 53.

103 Paragraph 53.

104 Dispute Regarding Navigational and Related Rights (Costa Rica v Nicaragua) [2009] ICJ Rep 213.

105 Paragraph 66.

106 Paragraphs 67–68.

107 Paragraph 71.

108 Paragraph 77.

109 Aegean Sea Continental Shelf Case, paragraph 77.

110 Paragraphs 64–65.

111 Paragraph 64.

112 Paragraph 67.

113 Dispute Regarding Navigational and Related Rights (Costa Rica v Nicaragua), paragraph 67.

114 [1998] WT/DS//56/AB/R, DSR 1998: VII, 2793-2798, paragraphs 126–134.

115 Paragraph 130.

116 Paragraph 130.

117 Articles 56, 61–62.

118 Paragraph 158.

119 Paragraph 112.

120 Paragraph 130.

121 Paragraphs 79–80.

122 Paragraph 80.

123 Scoppola v Italy (No 2) (2009) No 10249/03, paragraph 104.

124 Noting how the law uses analogy as a device for adapting to new technological developments.

125 Although, according to Study 2/3, to ensure quality, only 38 of them allow the submission of non-human sequences.

126 Study 2/3 reports that most nucleotide sequence data submissions to the INSDC come from major states like USA, China, Canada, and Japan, rather than developing states, which are usually perceived as the net providers of genetic resources. While this may mean that the former generally rely on their own stock of genetic resources, this should be considered alongside poor country of origin reporting and related tracing problems.

127 Study 2/3 attributes part of the problem to the fact that CBD parties normally issue documentation of prior informed consent (PIC) and mutually agreed terms (MAT) in pdf files, which technically cannot be linked to INSDC entries. Despite that, the INSDC submission form, itself, does not have PIC or MAT metadata field. However, Study 2/3 notes that the EMBL-EBI advises users of its services to ensure that data usage does not violate the intellectual property of third parties or their ABS rights relating to biodiversity.

128 Study 2/3 reports that the INSDC spends about $US50 million annually on maintenance, but has, so far, not charged any rent for access to its databases. How long this policy will continue, especially on the part of other databases that rely on the INSDC, is uncertain. As the Study notes, some databases have collapsed due to inability to meet maintenance costs.

129 A free INSDC service that enables users to download whole nucleotide sequence dataset as individual files, instead of visiting individual web pages.

130 See Preambles 20, 22 and articles 1, 15(2), CBD 1992; article 8, Nagoya Protocol 2010.
